# CDK4/6 Inhibitors in the First-Line Treatment of Postmenopausal Women with HR+/HER2− Advanced or Metastatic Breast Cancer: An Updated Network Meta-Analysis and Cost-Effectiveness Analysis

**DOI:** 10.3390/cancers15133386

**Published:** 2023-06-28

**Authors:** Ni Zeng, Jiaqi Han, Zijian Liu, Jinlan He, Kun Tian, Nianyong Chen

**Affiliations:** 1 Department of Head and Neck Oncology, Cancer Center and State Key Laboratory of Biotherapy, West China Hospital, Sichuan University, Chengdu 610041, China; 2 Department of Radiation Oncology, Cancer Center and State Key Laboratory of Biotherapy, West China Hospital, Sichuan University, Chengdu 610041, China; 3 Department of Andrology, Sichuan Human Sperm Bank, West China Second University Hospital, Sichuan University, Chengdu 610041, China; 4 Key Laboratory of Birth Defects and Related Diseases of Women and Children, Sichuan University, Ministry of Education, Chengdu 610041, China

**Keywords:** cost-effectiveness, network meta-analysis, HR+/HER2− advanced or metastatic breast cancer, CDK4/6 inhibitors, palbociclib, ribociclib, abemaciclib, letrozole, anastrozole

## Abstract

**Simple Summary:**

Recently updated results of clinical studies have confirmed that cyclin-dependent kinase 4/6 (CDK4/6) inhibitors such as palbociclib (Palbo), ribociclib (Ribo), and abemaciclib (Abem) plus letrozole/anastrozole (NSAI) significantly prolong survival compared to placebo plus NSAI in the first-line treatment of postmenopausal women with hormone receptor-positive (HR+) and human epidermal growth factor receptor-2 negative (HER2−) advanced or metastatic breast cancer (ABC). However, the high cost of CDK4/6 inhibitors imposes a huge financial burden on patients and healthcare systems. We conducted a network meta-analysis (NMA) and cost-effectiveness analysis (CEA) combined study to compare the effectiveness and cost-effectiveness of CDK4/6 inhibitors in HR+/HER2− ABC from the perspective of payers in China. Our study indicated that Abem + NSAI was cost-effective in China as the first-line treatment for postmenopausal women with HR+/HER2− ABC, owing to its better clinical efficacy and lower price. However, the Palbo + NSAI and Ribo + NSAI groups were not cost-effective unless adjusting drug prices to 50% or 10% of current prices ($320.67 per cycle or $264.60 per cycle).

**Abstract:**

(1) Background: This study aimed to conduct a NMA and CEA combined study to compare the effectiveness and cost-effectiveness of different CDK4/6 inhibitors (Abem, Palbo, and Ribo) plus NSAI with placebo plus NSAI in the first-line treatment of postmenopausal women with HR+/HER2− ABC from the perspective of payers in China. (2) Methods: Studies which evaluated CDK4/6 inhibitors plus NSAI for HR+/HER2− ABC were searched. A Bayesian NMA was carried out and the main outcomes were the hazard ratios (HRs) of overall survival (OS) and progression-free survival (PFS). The costs and efficacy of first-line therapies for HR+/HER2− ABC were evaluated using the Markov model. The main outcomes in the CEA were incremental cost–utility ratios (ICURs), incremental monetary benefit (INMB), and incremental net-health benefit (INHB). The robustness of the model was assessed by one-way, three-way, and probabilistic sensitivity analyses. Then, we further simulated the impact of different prices of CDK4/6 inhibitors on the results. (3) Results: Seven studies involving 5347 patients were included in the NMA. The three first-line CDK4/6 inhibitors plus NSAI groups provided significant PFS and OS superiority to NSAI alone. Abem + NSAI represented a significant statistical advantage onPFS (HR 0.74, 95% CI 0.61–0.90, *p* = 0.009) and indicated a trend of being the best OS benefit compared to the placebo + NSAI group (HR 0.89, 95% CI 0.72–1.08). The Abem + NSAI, Palbo + NSAI, and Ribo + NSAI groups resulted in additional costs of $12,602, $20,391, and $81,258, with additional effects of 0.38, 0.31, and 0.30 QALYs, respectively, leading to an ICUR of $33,163/QALY, $65,777/QALY, and $270,860/QALY. Additional pairwise comparisons showed that Abem + NSAI was the only cost-effective option in three CDK4/6 inhibitors plus NSAI groups at a willingness-to-pay (WTP) of $38,029/QALY. The sensitivity analyses showed that the proportion of receiving subsequent CDK4/6 inhibitors and the cost of Abem significantly influenced the results of Abem + NSAI compared with placebo + NSAI. (4) Conclusion: From the perspective of Chinese payers, Abem + NSAI was a cost-effective treatment option compared with placebo + NSAI at the WTP of $38,029/QALY, since only the ICUR of $33,163/QALY of Abem + NSAI was lower than the WTP of $38,029/QALY in China (2022). The Palbo + NSAI and Ribo + NSAI groups were not cost-effective unless drug prices were adjusted to 50% or 10% of current prices ($320.67 per cycle or $264.60 per cycle). (5) Others: We have prospectively registered the study with the PROSPERO, and the PROSPERO registration number is CRD42023399342.

## 1. Introduction

Breast cancer is the primary disease burden and the leading cause of cancer-associated mortality in women worldwide. According to the latest GLOBOCAN 2020 estimates, 2.6 million new breast cancer cases and 0.7 million breast cancer-associated deaths have occurred in 2020 [1]. An estimated 42,000 new cases of breast cancer are diagnosed in China annually. The most common molecular subtypes of breast cancer are the hormone receptor-positive (HR+) and human epidermal growth factor receptor-2 negative (HER2−) breast cancers [2]. Endocrine therapy is the main treatment for these patients. However, most patients will suffer clinical resistance to endocrine therapy, eventually leading to disease progression. The use of cyclin-dependent kinase 4/6 (CDK4/6) inhibitors has changed the clinical treatment paradigm for HR+/HER2− breast cancer [3].

CDK4/6 inhibitors work by inhibiting the phosphorylation of tumor suppressor retinoblastoma protein by preventing CDK4/6 from binding to cyclin D, therefore preventing cells from proliferating through the G1/S checkpoint [4]. At present, there are three CDK4/6 inhibitors that have been approved by the FDA and recommended in the NCCN (National Comprehensive Cancer Network) Clinical Practice Guidelines in Oncology, palbociclib (Palbo), ribociclib (Ribo), and abemaciclib (Abem) [5]. As well, dalpiciclib, a new CDK4/6 inhibitor, has been marketed in China. The latest studies for the first-line treatment of HR+/HER2− advanced breast cancer (ABC) based on these CDK4/6 inhibitors include the PALOMA series [6,7,8], the MONARCH series [5], the MONALEESA series [9], and the DAWNA series [10], most of which have achieved positive results.

Palbo, as the first CDK4/6 inhibitor, has the richest clinical data, including several RCTs (Randomized Controlled Trials) and real-world studies (RWS). Based on PALOMA-1, both of the phase 3 trials PALOMA-2 and PALOMA-4 reported significantly prolonged median progression-free survival (PFS) with first-line Palbo plus letrozole compared to placebo plus letrozole in postmenopausal women with HR+/HER2− ABC [6,11]. However, the updated data of PALOMA-2 in the ASCO of 2022 showed that patients receiving Palbo plus letrozole had numerically but not statistically significant longer overall survival (OS) compared with the placebo plus letrozole (51.6 vs. 44.6 months, HR = 0.869, 95% CI 0.706–1.069) [12]. The real-world research trial, P-reality X, supplemented the meaningful OS data of Palbo plus letrozole/anastrozole (NSAI) (49.1 vs. 43.2 months, HR = 0.76, 95% CI, 0.65–0.87, *p* < 0.0001) [13]. MONALEESA-2 demonstrated a better median PFS for Ribo plus letrozole as compared to placebo plus letrozole (25.3 vs. 16.0 months, HR = 0.568, 95% CI = 0.457–0.704, *p* < 0.0001) and the OS data were published in 2022 (63.9 vs. 51.4 months, HR = 0.76, 95% CI 0.63–0.93, *p* < 0.0001) [14,15]. The final PFS analysis of MONARCH 3 confirmed that Abem plus letrozole significantly improved the PFS compared to placebo plus letrozole (28.18 vs. 14.76 months, HR = 0.54, 95% CI = 0.418–0.698, *p* < 0.0001) [16]; the updated data of OS were announced at the ESMO congress of 2022 (67.1 vs. 54.5 months, HR= 0.754, 95% CI 0.584–0.974, *p* = 0.0301) [17], for which follow-up is ongoing for final OS analysis (expected in 2023). Further, the PFS data of DAWNA-2, a phase 3 clinical study comparing dalpiciclib plus NSAI with placebo + NSAI, were first reported in the ESMO Congress of 2022 (30.6 vs. 19.4 months, HR = 0.52, 95% CI 0.36–0.75) [10].

In summary, CDK4/6 inhibitors combined with NSAI have shown great efficacy in the treatment of advanced breast cancer. However, the lack of head-to-head clinical trials makes it difficult to directly compare the effectiveness of different CDK4/6 inhibitors. Network meta-analysis (NMA) is a useful method for comparing efficacy and for obtaining relative rankings in several competing treatments by combining direct evidence from head-to-head RCTs and indirect evidence from within a network. Apart from their effectiveness, despite the substantial clinical benefit, CDK4/6 inhibitors are expensive and place a heavy financial burden on patients and the society as a whole. Economic evaluations of CDK4/6 inhibitors plus endocrine therapy are urgently required to find better therapy regimens and provide evidence for government health insurance decision-making. Cost-effectiveness analysis (CEA) is the most prevalent method for economic evaluation which assesses medically relevant outcomes in naturally occurring health-related units such as life-years (LYs) gained. Since the publication of the latest survival results, there has been no new CEA study published from the perspective of China. 

Therefore, this study aimed to conduct a NMA and CEA of CDK4/6 inhibitors (Abem, Palbo, Ribo) to compare the effectiveness and cost-effectiveness of CDK4/6 inhibitors (Abem, Palbo, Ribo) in HR+/HER2− ABC from the perspective of payers in China. Our analysis can provide a valuable reference for the selection of optimal CDK4/6 inhibitor treatment for patients with HR+/HER2− ABC.

## 2. Materials and Methods

Our NMA and CEA were in accordance with the Preferred Reporting Items for Systematic Reviews and Meta-Analysis (PRISMA) extension statement and the consolidated health economic evaluation reporting standards (CHEERS) statement [18] (Supplementary Tables S1 and S2). We have prospectively registered the study with PROSPERO, and the PROSPERO registration number is CRD42023399342.

### 2.1. Network Meta-Analysis (NMA) 

#### 2.1.1. Study Eligibility and Selection 

We developed searches to identify eligible studies published in the PubMed, EMBASE, Web of Science, Cochrane Library, and ClinicalTrials.gov databases that compare CDK4/6 inhibitors plus endocrine treatments with endocrine treatments for patients with previously untreated HR+/HER2− ABC, with the deadline being up to 10 February 2023. We also searched the proceedings of the annual oncology conferences presented in 2017–2022 for the latest outcomes, including the American Society of Clinical Oncology (ASCO), the European Society of Medical Oncology (ESMO), and the American Association for Cancer Research (AACR). The literature search strategies were developed using medical subject headings (MeSH) and keywords; details can be found in Supplementary Table S3. The eligible literature met the following criteria: (1) RCTs or high-quality RWSs; (2) compared CDK4/6 inhibitors plus NASI with placebo + NSAI for patients with previously untreated HR+/HER2− ABC; (3) the primary outcomes were OS or PFS; and (4) each CDK4/6 inhibitor has at least one study that provided complete surviving data, including OS data and PFS data. 

#### 2.1.2. Data Collection and Assessment of the Risk of Bias 

We have considered pivotal randomized controlled trials of Palbo + NSAI, Ribo + NSAI, and Abem + NSAI compared with placebo + NSAI for the first-line treatment of postmenopausal women with HR+/HER2− ABC. The last available update of each trial was considered as the source. Two authors (N Zeng and JQ Han) performed, independently, the study selection, data extraction, and quality assessment according to the standard PRISMA statement. Detailed basic information about the clinical trial was collected and is shown in Supplementary Table S4. The efficacy clinical data were extracted in the NMA, including hazard ratio (HR) with corresponding 95% confidence interval (CI) of PFS and OS in each clinical trial. The risks of bias in clinical trials were evaluated using the Cochrane Risk of Bias Tool 2.0.

#### 2.1.3. Statistical Analysis 

We select HRs of the OS and PFS as the primary outcomes of our NMA. A Bayesian framework using Markov chain Monte Carlo methods by the Open BUGS software (version 3.2.3; Available: http://www.openbugs.net/w/Downloads, accessed on 20 October 2022) was used to make the direct and indirect comparisons. Both the fixed-effects and random-effects models were fitted, and the final model was chosen as that with the smallest deviance information criterion (DIC) value.

### 2.2. Cost-Effectiveness Analysis (CEA)

#### 2.2.1. Overview

We constructed a Markov model to compare the three CDK4/6 inhibitors (Palbo, Ribo, and Abem) plus NSAI compared with placebo + NSAI. We choose total costs, quality-adjusted life-years (QALYs), life-years (LYs), incremental cost-utility ratios (ICURs), incremental cost-effectiveness ratios (ICERs), incremental monetary benefit (INMB), and incremental net-health benefit (INHB) as the main outcomes in our CEA. We used three times the per-capita gross domestic product (GDP) ($38,029, in 2022) as a threshold for willingness-to-pay (WTP) based on China’s pharmacoeconomic assessments guidelines and the World Health Organization (WHO) [19]. Drug costs, major adverse events costs, subsequent therapies, supportive care costs, follow-up costs (including imaging and laboratory test), and end-of-life care were considered direct medical costs from the perspective of patients in China. The primary outcome was determined based on the comparison of the ICUR and WTP between each two groups.

#### 2.2.2. Base-Case Analyses Population and Interventions 

The Markov model was conducted based on the clinical studies selected in the NMA, including PALOMA-1, PALOMA-2, PALOMA-4, P-reality X, MONALEESA-2, MORNARCH 3, and MORNACH plus [5,9,14,16,20,21]. All the studies compared CDK4/6 inhibitors (Palbo, Ribo, and Abem) plus NSAI with placebo + NSAI in the first-line therapy of postmenopausal women with HR+/HER2− ABC. The MONARCH plus study included both NSAI and fulvestrant as endocrine therapy, although only NSAI data were used in our study.

Letrozole or anastrozole were administered at a dosage of 2.5 mg or 1 mg once daily on a continuous schedule. Palbo or Ribo were administered at a dosage of 125 mg or 600 mg once daily for 3 weeks followed by 1 week off in 28-day cycles. Abem was administered at a dosage of 150 mg twice daily on a continuous schedule. The treatment continued until disease progression, unacceptable toxic effects, or death [6,9,11,12,13,14,15,16,17]. A mean weight of 65 kg and a mean body surface area of 1.72 m^2^ were used to calculate drug dosages [22,23,24,25].

#### 2.2.3. Model Structures 

**Figure 1:**
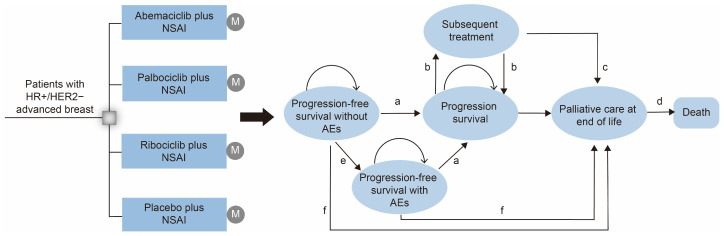
Markov model structure for cost-effectiveness analysis. Markov model depicting the four first-line treatment arms and the transition between Markov states, illustrating the disease development process of HR+/HER2− ABC. (**a**) Progression-free survival without AEs; (**b**) progression survival; (**c**) overall survival after progression; (**d**) death; (**e**) progression-free survival with AEs; (**f**) first-line overall survival. NSAI, letrozole/anastrozole; AEs, adverse events.

In this study, the Markov model was developed to evaluate the cost-effectiveness of treatment with CDK4/6 inhibitors + NSAI or placebo + NSAI for postmenopausal women with HR+/HER2− ABC using TreeAge Pro 2020 software (TreeAge Software Inc., Williamstown, MA, USA) from the perspective of payers in China. We simulated a population similar to the PALOMA-1, PALOMA-2, PALOMA-4, P-reality X, MONALEESA-2, MORNARCH 3, and MORNACH plus trials (Supplementary Table S5) [5,9,14,16,20,21]. Eligible patients were randomly divided into four groups: (1) Palbo + NSAI group; (2) Ribo + NSAI group; (3) Abem + NSAI group; and (4) placebo + NSAI group. For each treatment arm, the Markov model consisted of three mutually exclusive states: progression-free survival (PFS), progressive disease (PD), and death (Figure 1). In the model, the proportion of patients in each health state at each time point was determined from OS and PFS curves. The model terminated once all patients were in the death Markov state. The model cycle length was 4 weeks, consistent with a clinical treatment cycle. A lifelong time horizon was adapted to capture related costs and outcomes. A 3% annual discount rate was performed for cost and survival simulation in all groups. A half-cycle correction was applied equally to each model. 

#### 2.2.4. Transition Probabilities 

The transition probabilities between different Markov states (PFS, PD, and death) of the survival model were calculated to simulate the whole progress of the disease. GetData Graph Digitizer software version 2.20 was used to extract the survival time-to-event data of the placebo + NSAI group from the Kaplan–Meier curves of the PALOMA-1, PALOMA-2, PALOMA-4, P-reality X, MONALEESA-2, MORNARCH 3, and MORNACH plus trials following the procedure described by Hoyle et al. [26]. Subsequently, the flexible parametric survival models, including the Exponential, Weibull, Log-logistic, Lognormal, and Gompertz models, were used to reconstruct the survival data by the R software. The Log-logistic model provided good fitting results for all Kaplan–Meier curves according to visual fit, clinical rationality, and statistical fit. The detailed model selection procedure was described in Supplementary Table S6 and Figure S1. In addition, given the absence of head-to-head clinical trial data, the results of this NMA were used to obtain the direct and indirect survival comparison data among the three CDK4/6 inhibitor combination groups that were input into our Markov model. The disease-cause mortality rate was estimated from the OS curves, while mortality from other causes was estimated from the life table in China (Supplementary Table S7) [27]. 

#### 2.2.5. Costs and Utilities Inputs

Only direct medical costs were incorporated into our model, as follows: drug costs, severe adverse events costs (grade 3 or 4 AEs), subsequent therapies costs, supportive care costs, follow-up costs (including imaging and laboratory tests), and end-of-life care costs. The unit cost of this section is based on previous studies [22,23,24,25]. Direct unit cost data were extracted from hospital accounting databases of the local database of China in Chinese yuan (CNY) and reported in 2023 US$ ($1 = 6.7602 CNY, 18 January 2023) [28]. Unit drug doses, routes of administration, frequency of adverse events, and proportion of subsequent therapies in the four groups were based on the PALOMA-2, MONALEESA-2, and MORNARCH-3 clinical studies (Table 1). The costs of management of grade 3 or 4 AEs were derived from the local database of China (Table 2). The subsequent treatment options were selected in our analysis according to the selected clinical trials and NCCN guidelines (Table 1) [9,20,21,29,30]. The detailed calculation process is described in Supplementary Table S8. The mean utilities estimated for PFS and PD states were from the previously published literature (Table 2) [25]. 

#### 2.2.6. Sensitivity Analyses

We performed a series of one-way, three-way, and probabilistic sensitivity analyses to ascertain the robustness of the model and the variable uncertainty of the results. One-way sensitive analysis was used to identify the sensitive input factors based on distributions corresponding to the ranges of variation. Considering that the costs of Palbo, Ribo, and Abem might change simultaneously, three-way sensitivity analysis provided a complement to one-way sensitivity analysis to assess the influence of CDK4/6 inhibitors’ costs on the main outcomes. Moreover, probabilistic sensitivity analysis was conducted to assess the robustness that varied all variables simultaneously by 10,000 iterations of Monte Carlo simulation.

## 3. Results

### 3.1. Network Meta-Analysis (NMA)

We identified 891 records through database searching and an additional 3 records through other sources. After the duplicates were eliminated and the articles were screened, five phase III RCTs, one phase II clinical study, and one high-quality RWS, including 5347 patients, were considered (including 3138 patients taking CDK4/6 inhibitors plus NSAI and 2209 patients taking placebo + NSAI). The main reported outcomes of the analyzed studies are reported in Table 1, Table 2 and Table 3.

**Figure 2:**
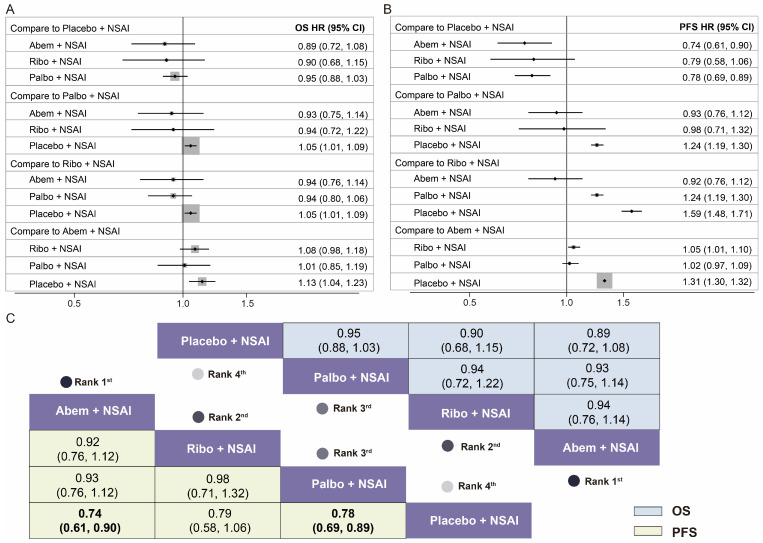
Efficacy results of the Bayesian network meta-analysis in the four first-line treatment groups for patients with HR+/HER2− ABC. (**A**) Forest plot of hazard ratios and 95% confidence intervals for overall survival in pairwise comparisons of the four first-line treatment groups; (**B**) forest plot of hazard ratios and 95% confidence intervals for progression-free survival in pairwise comparisons of the four first-line treatment groups; (**C**) hazard ratios, 95% confidence intervals, and ranking for overall survival (upper triangle in blue) and progression-free survival (lower triangle in yellow) of the network meta-analysis in the four first-line treatment groups. Palbo + NSAI, palbociclib plus letrozole/anastrozole; Ribo + NSAI, ribociclib plus letrozole/anastrozole; Abem + NSAI, abemaciclib plus letrozole/anastrozole; placebo + NSAI, placebo plus letrozole/anastrozole.

Seven studies (PALOMA-1, PALOMA-2, PALOMA-4, P-reality X, MONALEESA-2, MORNARCH-3, and MORNACH plus), involving 5347 patients, were included in the NMA, and the searching flow chart is shown in Supplementary Figure S2. The network plot is detailed in Supplementary Figure S3. The results of the risk of bias assessment suggested a low bias risk in these seven studies (Supplementary Figure S4). A fixed-effects model was chosen based on the lower value of DIC. The primary outcomes in the NMA were the HRs of OS and PFS, as shown in Figure 2. Concerning the primary endpoints of the HRs of PFS, combination treatments with two CDK4/6 inhibitors were significantly better than placebo + NSAI, including Abem + NSAI (HR 0.74, 95% CI 0.61–0.90, *p* = 0.009) and Palbo + NSAI (HR 0.78, 95% CI 0.69–0.89, *p* = 0.012). Between the Ribo + NSAI and placebo + NSAI groups, there was no substantial difference in the PFS, but a clear trend was shown (HR 0.79, 95% CI 0.58–1.06). Although the results showed no significant efficacy for the OS benefit, there was a trend toward a better OS in the three CDK4/6 inhibitors combination options compared with placebo + NSAI (Abem + NSAI vs. placebo + NSAI: HR 0.89, 95% CI 0.72–1.08; Ribo + NSAI vs. placebo + NSAI: HR 0. 90, 95% CI 0.68–1.15; Palbo + NSAI vs. placebo + NSAI: HR 0.95, 95% CI 0.88–1.03). Additionally, among the combination therapies, better OS (HR 0.89, 95% CI 0.72–1.08) and PFS (HR 0.74, 95% CI 0.61–0.90, *p* = 0.009) benefits were both obtained from the Abem + NSAI group (Figure 2).

### 3.2. Cost-Effectiveness Analysis (CEA)

#### 3.2.1. Baseline Results

The probabilities of Markov states corresponding to the different cycles of treatment for patients are detailed in Supplementary Figure S5. The baseline results with a lifetime horizon, including the total costs and effectiveness for each treatment group, are presented in Table 3. Treatment with placebo + NSAI resulted in average lifetime costs of $70,743 and 3.78 QALYs. Overall, compared with the placebo + NSAI group, the Abem + NSAI group was the only cost-effective strategy among the three CDK 4/6 inhibitor combination groups, with an additional 0.38 QALYs and an incremental cost of $12,602, resulting in an ICUR of $33,163/QALY. In addition, the INHB was 0.05 QALYs, and the INMB was $1849 at the WTP threshold of $38,029/QALY. The Palbo + NSAI and Ribo + NSAI groups were not cost-effective compared to the placebo + NSAI group. In specific, the Palbo + NSAI group gained an additional 0.31 QALYs with $20,397 more in costs (ICUR = $65,777/QALY, INHB = −0.23 QALYs); the Ribo + NSAI group gained an additional 0.30 QALYs with an additional $81,258 (ICUR = $270,860/QALY, INHB = −1.84 QALYs). To better ascertain the strategies that were more cost-effective in the three CDK4/6 inhibitors plus NSAI groups, additional results of the pairwise comparisons are presented in Table 4. Notably, the results indicated that Abem + NSAI was the cost-effective treatment strategy when compared to the Palbo + NSAI and Ribo + NSAI groups, respectively. 

#### 3.2.2. Sensitivity Analyses 

**Figure 3:**
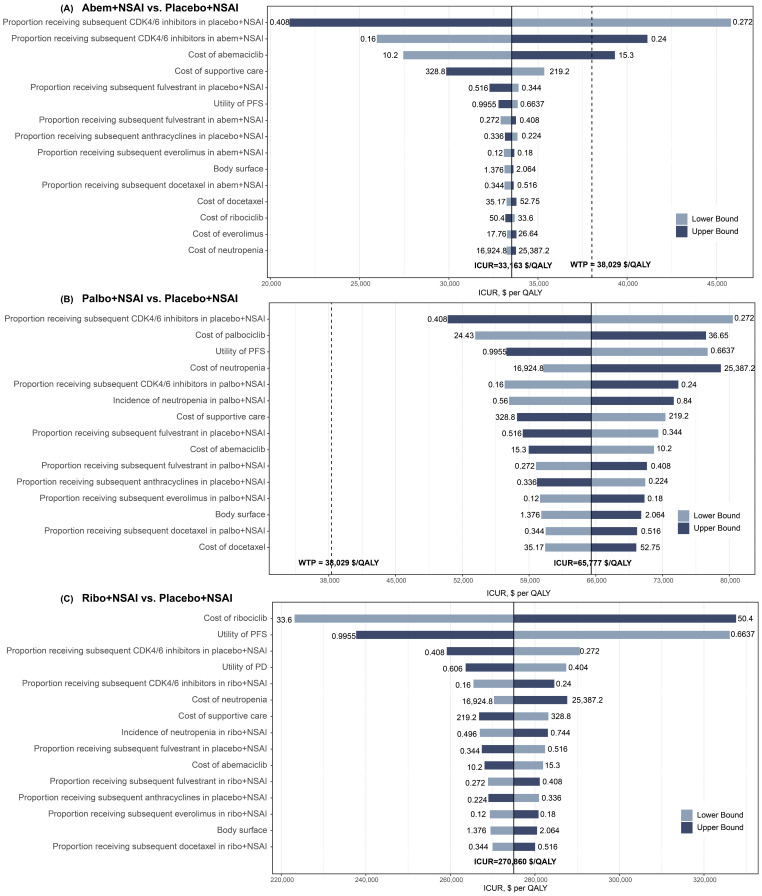
Tornado diagrams derived from the one-way sensitivity analyses. (**A**) Abem + NSAI vs. placebo + NSAI; (**B**) Palbo + NSAI vs. placebo + NSAI; (**C**) Ribo + NSAI vs. placebo + NSAI. The black solid line represents the ICURs. The dashed line represents the WTP threshold in China ($38,029/QALY).

**Figure 4:**
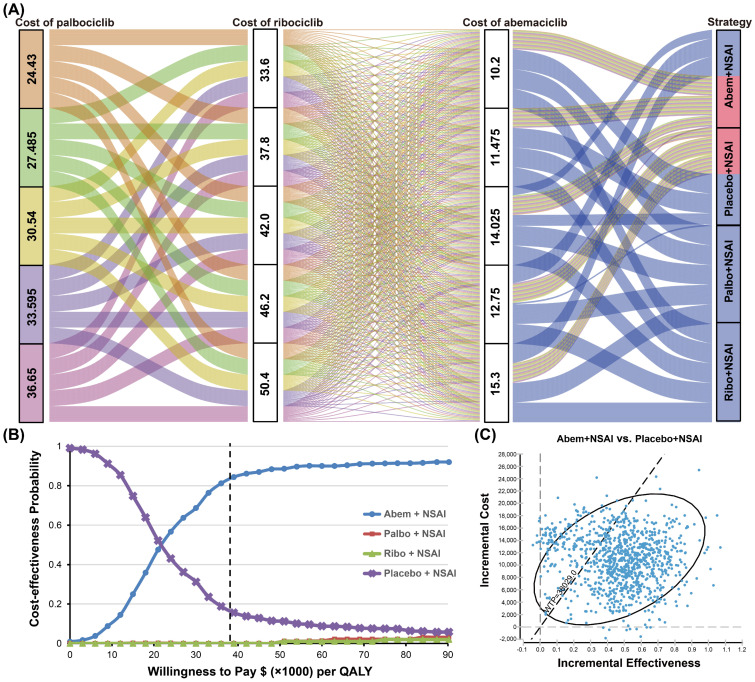
Results of three-way and probability sensitivity analyses. (**A**) Results of three-way sensitivity analyses. The Abem + NSAI group was the best strategy (red cell in strategy column) when the costs of palbociclib, ribociclib, and abemaciclib varied within a certain range. (**B**) Cost-effectiveness acceptability curves. The probabilities that the treatment option is cost-effective at different willingness-to-pay thresholds. (**C**) Probability sensitivity analysis scatter plot in the Abem + NSAI group compared to the placebo + NSAI group. Each point in the diagram represents a simulation result of 10,000 Monte Carlo simulations. The ellipse represents the 95% CI and the dotted line represents the WTP threshold in China ($38,029/QALY).

The pairwise one-way sensitivity analyses showed that some model variables had a significant influence on the results of Abem + NSAI compared with placebo + NSAI, which are presented in Figure 3. Varying the proportion of the received subsequent CDK4/6 inhibitors and the cost of Abem had a substantial impact on the outcomes of the model in the comparison across the Abem + NSAI and placebo + NSAI groups. Of note, when the proportion of receiving subsequent CDK4/6 inhibitors in the placebo + NSAI group decreased to 0.272, the proportion of receiving subsequent CDK4/6 inhibitors in the Abem + NSAI group increased to 0.24, and the costs of Abem increased to 15.3, the ICURs were higher than the WTP of $38,029/QALY (Figure 3A). Compared to the placebo + NSAI group, irrespective of the changes in the model parameters, the ICURs of the Palbo + NSAI and Ribo + NSAI groups were higher than the WTP, validating the robustness of our model (Figure 3B,C). The three-way sensitivity analyses indicated that, when the cost of Palbo, Ribo, and Abem changed simultaneously, the most cost-effective strategy was selected as being between the Abem + NSAI and placebo + NSAI groups. This result revealed that the prices of CDK4/6 inhibitors exert great influence on the main outcomes (Figure 4A). In the cost-effectiveness acceptability curves, the probabilities that the Abem + NSAI group was cost-effective increased as the WTP threshold increased (Figure 4B). The probability of Abem + NSAI being cost-effective was 81.3% at the WTP threshold of $38,029 per QALY (Figure 4C). The more detailed incremental cost-effectiveness scatterplots are shown in Supplementary Figure S6.

#### 3.2.3. Variations in the Cost of CDK4/6 Inhibitors

**Figure 5:**
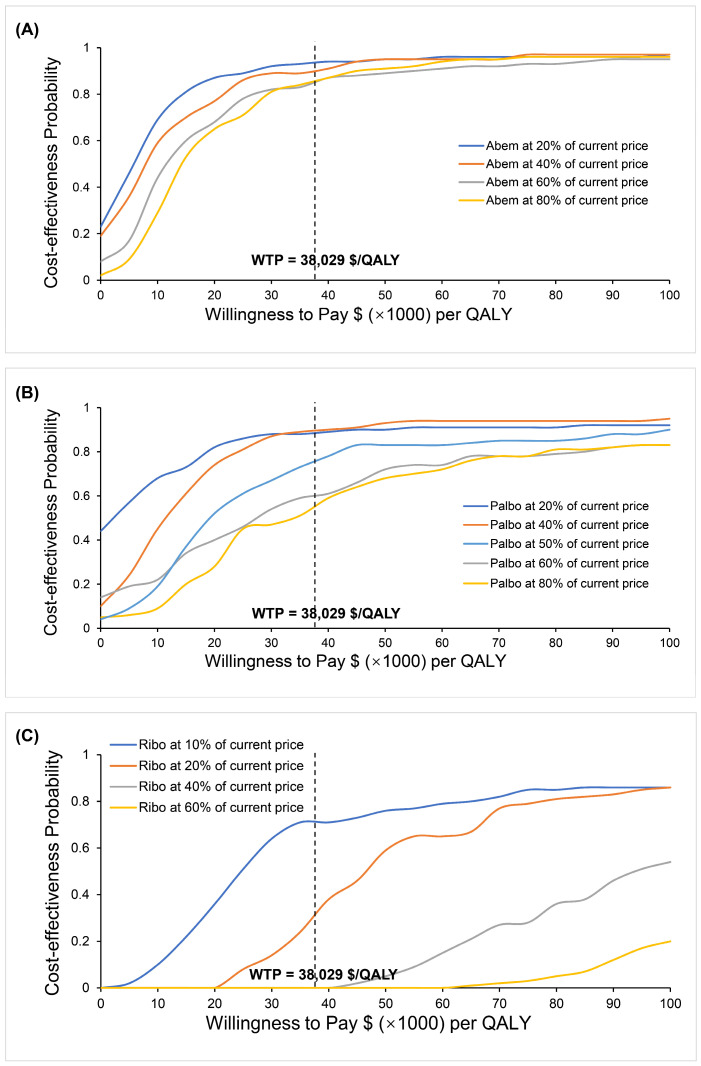
Results of simulating the impact of different prices on cost-effectiveness. (**A**) The cost-effectiveness probability of Abem + NSAI when the price of abemaciclib was 80%, 60%, 40%, and 20% of the current price, respectively. (**B**) The cost-effectiveness probability of Palbo + NSAI when the price of palbociclib was 80%, 60%, 50%, 40%, and 20% of the current price, respectively. (**C**) The cost-effectiveness probability of Ribo + NSAI when the price of ribociclib was 60%, 40%, 25%, 20%, and 10% of the current price, respectively.

The sensitivity analyses indicated that the prices of CDK4/6 inhibitors had a great impact on the results, so we further simulated the impact of different prices on the results. We simulated the unit cost of Abem at 80%, 60%, 40%, and 20% of the current price, respectively, which resulted in the change in the price having no obvious impact on the cost-effective benefit of the Abem + NSAI group. Palbo + NSAI will be cost-effective when the price of Palbo reduces to half of the current value ($320.67 per cycle). Ribo + NSAI could be a cost-effective strategy when the price of Ribociclib decreases to 10% of the current price ($264.60 per cycle) (Figure 5).

## 4. Discussion

Hormone receptor-positive breast cancer is the most common molecular subtype of cancer [2]. The emergence of CDK4/6 inhibitors has changed the prognosis of patients with hormone receptor-positive breast cancer, and their combination with endocrine therapy has been recommended as a first-line therapy in clinical guidelines [4,29]. However, in the absence of a direct comparison between CDK4/6 inhibitors (Abem, Palbo, and Ribo), there is no direct evidence available to help patients, clinicians, and policymakers assess which combination might be better. To provide a better reference for treatment options, we synthesized the latest evidence, performed an NMA, and constructed a Markov model to evaluate the cost and effectiveness among the first-line treatments of CDK4/6 inhibitors combined with NSAI in the first-line treatment for postmenopausal women with HR+/HER2− ABC. Of note, in addition to the three CDK4/6 inhibitors included in the study (Abem, Palbo, and Ribo), dalpiciclib achieved a significant improvement in PFS in both premenopausal and postmenopausal populations based on the preliminary results reported in the DAWNA-2 trial (30.6 vs. 19.4 months, HR = 0.52, 95% CI 0.36–0.75) [10]. Due to the immaturity of the OS data, we did not include dalpiciclib in our analysis.

Based on our results from the NMA, the three first-line CDK4/6 inhibitors plus NSAI provided PFS and OS superiority to placebo + NSAI. Among the three CDK4/6 inhibitors plus NSAI groups, Abem + NSAI represented a significant statistical advantage regarding PFS (HR 0.74, 95% CI 0.61–0.90, *p* = 0.009) and indicated a trend of having the best OS benefit (HR 0.89, 95% CI 0.72–1.08); however, the results of the OS should be interpreted with caution since no significant statistical difference was found.

The results of further CEA showed that, compared to the placebo + NSAI group, the Abem + NSAI, Palbo + NSAI, and Ribo + NSAI groups resulted in additional costs of $12, 602, $20,391, and $81,258, with the additional effects of 0.38, 0.31, and 0.30 QALYs, respectively, leading to ICURs of $33,163/QALY, $65,777/QALY, and $270,860/QALY. The above results demonstrate that, among the three first-line CDK4/6 inhibitors plus NSAI groups, only Abem + NSAI was more cost-effective compared to the placebo + NSAI for HR+/HER2− ABC from the perspective of Chinese payers. The same conclusion was also reflected in the values of the ICERs, INMBs, and INHBs. As a newly marketed targeted drug that has not yet been covered by national health insurance, the cost of Ribo is extremely high, which limits the cost-effectiveness of the Ribo combination strategy.

One-way sensitivity analyses demonstrated that, when the proportion of receiving subsequent CDK4/6 inhibitors in the placebo + NSAI group decreased to 0.272, the proportion of receiving subsequent CDK4/6 inhibitors in the Abem + NSAI group increased to 0.24, and the costs of Abem increased to 15.3, the ICURs were higher than the WTP of $38,029/QALY. The possible reasons for the proportion of subsequent CDK4/6 inhibitors and the cost of Abem influencing the outcomes were the relatively higher prices of CDK4/6 inhibitors and a small difference between the values of the ICUR and WTP, which led to the greater possibility that ICUR exceeds the WTP. Three-way sensitivity analyses indicated that, when the cost of Palbo, Ribo, and Abem changed simultaneously, the most cost-effective strategy was selected to be between the Abem + NSAI and placebo + NSAI groups. In the cost-effectiveness acceptability curves, the probability that the Abem + NSAI group was cost-effective increased as the WTP threshold increased. The sensitivity analyses indicated that the prices of CDK4/6 inhibitors had a great impact on the results, so we further simulated the impact of different prices on the results. The change in the price of Abem has no obvious impact on the cost-effective benefit of the Abem + NSAI group. Palbo + NSAI will be cost-effective when the price of Palbo reduces to half of the current value. Ribo + NSAI could be a cost-effective strategy when the price of Ribo decreases to 10% of the current price. The use of CDK4/6 inhibitors has been the first-line treatment paradigm for HR+/HER2− breast cancer. However, the affordability of drugs needs to be taken into account to avoid lower drug utilization due to the prices. From our results, the use of CDK4/6 inhibitors will be encouraging if the drug prices are lowered, as this could improve the affordability of the drug to patients and the government. This conclusion may guide future price adjustments for CDK4/6 inhibitors. 

Abem was found to be cost-effective in China, largely owing to its lower cost. Even though the interim OS results of MONARCH-3 did not reach statistical significance (HR = 0.754, 95% CI 0.584–0.974, *p* = 0.0301), its absolute value is also very impressive, reaching 67.1 months. We will conduct further analyses further in conjunction with dalpiciclib when the final data are published. Ribo has just been launched in China and is not included in the national medical insurance list, so its price is much higher than the other two drugs, which might be the reason that the Ribo combination treatment was the least cost-effective strategy. 

Most published CEA studies found that CDK4/6 inhibitors plus endocrine therapies were not cost-effective compared to endocrine therapy alone. A series of CEA for the two-drug comparison of Palbo and Ribo found that Ribo plus letrozole was more cost-effective than Palbo plus letrozole in the first-line treatment of postmenopausal women [24,25,31]. Some of the studies conducted the Markov model to evaluate the cost-effectiveness between Palbo + NSAI and placebo + NSAI, producing consistent results that Palbo + NSAI was not cost-effective compared with placebo + NSAI from the perspective of the United States, Switzerland, and Canada [32,33,34]. A CEA study by Wan, X et al. estimated the price of Ribo before the drug’s marketing in China, and found that Ribo + NSAI is cost-effective when Ribo costs less than $721 or $1170 per four weeks [35]. In a CEA published before Ribo was marketed in China for premenopausal women with HR+/HER2− breast cancer, Huang et al. showed that the additional use of Ribo was not cost-effective in the United States, while it could be cost-effective when the price was less than $31.74/200 mg in China (in the year 2018, the three-times-per-capita GDP was $29,383/QALY), which is close to our estimate [36]. The latest commentary article conducted a cost-effectiveness analysis of the application of the three CDK4/6 inhibitors (Palbo, Ribo, and Abem) from the perspective of Europe and the USA, and found that the three CDK4/6 inhibitors plus endocrine therapy were not more cost-effective than endocrine therapy alone [37,38]. 

To the best of our knowledge, our study has several strengths. First, this study was the first CEA including all of the CDK4/6 inhibitors used in the first-line treatment of women with HR+/HER2− ABC in China. The sample size was large, and the analysis was comprehensive. This study included not only PALOMA-1-, PALOMA-2-, MONALEESA-2-, and MONARCH-3-enrolled patients, who are mainly from the USA, but also the PALOMA-4 and MONARCH plus studies, which included patients mainly from Asia. Therefore, this study’s result can be more confidently generalized to the Chinese population. Furthermore, survival data in the RCTs are becoming increasingly mature, and the OS and PFS data used in our study were both updated. On 18 February 2023, Ribo was officially launched in China, and the other two drugs (Palbo and Abem) were entered into the Chinese national medical insurance list. The costs data used in our study were also the latest. Besides, compared with previous CEA studies, our study included more comprehensive methods such as NMA, three-way sensitivity analysis, and all-cause background mortality, and further simulated the impact of different prices on the results. 

This analysis also had several limitations. First, we conducted a NMA to integrate the survival data because of a lack of direct comparisons among the three CDK4/6 inhibitors plus NSAI groups in clinical trials. The level of evidence from the NMA was lower than the RCTs. However, it is nearly impossible to compare the three treatments by RCTs; moreover, we did not find a significant heterogeneity or risk of bias in the NMA. Second, due to the incomplete QOL data in the RCTs, utilities used in our model were derived from the published literature [24,25]. Additional sensitivity analyses indicated that the input utilities had a minimal impact on our results. Third, only grade 3 or higher AEs were included in our Markov model, which might underestimate the cost of AE management. However, mild-grade AEs require almost no management, and further sensitivity analyses demonstrated that the effect on the conclusion was small. Finally, the interim OS data of MONARCH-3 did not reach statistical significance, but their absolute value is also very impressive. The updated data published in 2022 were used in this analysis and, when the final data are published, we will update our results.

## 5. Conclusions

In conclusion, our NMA and CEA combined analysis indicated that the three first-line CDK4/6 inhibitors (Palbo, Ribo, and Abem) plus NSAI therapies provided survival benefits on OS and PFS over placebo + NSAI for patients with HR+/HER2− ABC. Abem + NSAI displayed a significant statistical advantage over PFS and indicated a trend of having the best OS benefit compared to the placebo + NSAI group (PFS:HR 0.74, 95% CI 0.61–0.90, *p* = 0.009; OS: HR 0.89, 95% CI 0.72–1.08). Only Abem + NSAI was cost-effective compared to placebo + NSAI at the WTP of $38,029/QALY from the Chinese payers’ perspective, because only the ICUR of $33,163/QALY of Abem + NSAI was lower than the WTP of $38,029/QALY. However, the Palbo + NSAI and Ribo + NSAI groups were not cost-effective at the current price, unless adjusting drug prices to 50% or 10% of their current prices ($320.67 per cycle or $264.60 per cycle).

## Figures and Tables

**Table 1:** Model parameters of clinical data: baseline values, ranges, and distributions.

Variable	Baseline Value (Range)	Reference	Distribution
Placebo + NSAI OS survival model	λ = 0.001027, γ = 1.814	[6,9,11,12,13,14,15,16,17]	-
Placebo + NSAI PFS survival model HR for OS	λ = 0.045913, γ = 1.008831	[6,9,11,12,13,14,15,16,17]	-
Abem + NSAI vs. Placebo + NSAI	0.89 (0.72–1.08)	[6,9,11,12,13,14,15,16,17]	-
Ribo + NSAI vs. Placebo + NSAI	0.90 (0.68–1.15)	[6,9,11,12,13,14,15,16,17]	-
Palbo + NSAI vs. Placebo + NSAI HR for PFS	0.95 (0.88–1.03)	[6,9,11,12,13,14,15,16,17]	-
Abem + NSAI vs. Placebo + NSAI	0.74 (0.61–0.90)	[6,9,11,12,13,14,15,16,17]	-
Ribo + NSAI vs. Placebo + NSAI	0.79 (0.58–1.06)	[6,9,11,12,13,14,15,16,17]	-
Palbo + NSAI vs. Placebo + NSAI	0.78 (0.69–0.89)	[6,9,11,12,13,14,15,16,17]	-
Background mortality rate	Age specific	[27]	-
Palbo + NSAI, Ribo + NSAI, and Abem + NSAI Subsequent therapy proportion			
Exemestane	0.260 (0.208–0.312)	[6,9,11,12,13,14,15,16,17]	Beta (407, 1159)
NSAI	0.180 (0.144–0.216)	[6,9,11,12,13,14,15,16,17]	Beta (282, 1284)
Fulvestrant	0.340(0.272–0.408)	[6,9,11,12,13,14,15,16,17]	Beta (532, 1034)
Tamoxifen	0.140 (0.112–0.168)	[6,9,11,12,13,14,15,16,17]	Beta (219, 1347)
Everolimus	0.150 (0.120–0.180)	[6,9,11,12,13,14,15,16,17]	Beta (234, 1332)
Anthracyclines	0.190 (0.152–0.228)	[6,9,11,12,13,14,15,16,17]	Beta (297, 1269)
Capecitabine	0.340 (0.272–0.408)	[6,9,11,12,13,14,15,16,17]	Beta (532, 1034)
Gemcitabine	0.050 (0.040–0.060)	[6,9,11,12,13,14,15,16,17]	Beta (78, 1488)
Docetaxel	0.430 (0.344–0.516)	[6,9,11,12,13,14,15,16,17]	Beta (673, 893)
Vinorelbine	0.150 (0.120–0.180)	[6,9,11,12,13,14,15,16,17]	Beta (234, 1332)
CDK4/6 inhibitors	0.200 (0.160–0.240)	[6,9,11,12,13,14,15,16,17]	Beta (313, 1253)
Placebo + NSAI Subsequent therapy proportion			
Exemestane	0.350 (0.280–0.420)	[6,9,11,12,13,14,15,16,17]	Beta (375, 697)
NSAI	0.250 (0.200–0.300)	[6,9,11,12,13,14,15,16,17]	Beta (268, 804)
Fulvestrant	0.430 (0.344–0.516)	[6,9,11,12,13,14,15,16,17]	Beta (460, 612)
Tamoxifen	0.220 (0.176–0.264)	[6,9,11,12,13,14,15,16,17]	Beta (236, 835)
Everolimus	0.170 (0.136–0.204)	[6,9,11,12,13,14,15,16,17]	Beta (182, 890)
Anthracyclines	0.280 (0.224–0.336)	[6,9,11,12,13,14,15,16,17]	Beta (300, 772)
Capecitabine	0.420 (0.336–0.504)	[6,9,11,12,13,14,15,16,17]	Beta (450, 622)
Gemcitabine	0.100 (0.880–0.120)	[6,9,11,12,13,14,15,16,17]	Beta (107, 965)
Docetaxel	0.390 (0.312–0.468)	[6,9,11,12,13,14,15,16,17]	Beta (419, 653)
Vinorelbine	0.080 (0.064–0.096)	[6,9,11,12,13,14,15,16,17]	Beta (86, 986)
CDK4/6 inhibitors	0.340 (0.272–0.408)	[6,9,11,12,13,14,15,16,17]	Beta (364, 708)
Palbo + NSAI AEs incidence (Grade 3 or higher)			
Anemia	0.053 (0.042–0.064)	[6,11,12,13]	Beta (37, 659)
Thrombocytopenia	0.032 (0.025–0.038)	[6,11,12,13]	Beta (22, 674)
Neutropenia	0.700 (0.560–0.840)	[6,11,12,13]	Beta (487, 209)
Leukopenia	0.267 (0.214–0.320)	[6,11,12,13]	Beta (186, 510)
Nausea	0.004 (0.003–0.005)	[6,11,12,13]	Beta (3, 693)
Diarrhea	0.016 (0.013–0.019)	[6,11,12,13]	Beta (11, 685)
Fatigue	0.022 (0.017–0.026)	[6,11,12,13]	Beta (15, 681)
Hepatobiliary toxicity	0.020 (0.016–0.024)	[6,11,12,13]	Beta (14, 682)
Infection	0.016 (0.013–0.019)	[6,11,12,13]	Beta (11, 685)
Vomiting	0.0030 (0.0024–0.0036)	[6,11,12,13]	Beta (2, 694)
Ribo + NSAI AEs incidence (Grade 3 or higher)			
Anemia	0.024 (0.019–0.029)	[9,14,15]	Beta (8, 326)
Neutropenia	0.620 (0.496–0.744)	[9,14,15]	Beta (207, 127)
Leukopenia	0.213 (0.170–0.256)	[9,14,15]	Beta (71, 263)
Nausea	0.024 (0.019–0.029)	[9,14,15]	Beta (8, 326)
Diarrhea	0.024 (0.019–0.029)	[9,14,15]	Beta (8, 326)
Fatigue	0.030 (0.024–0.036)	[9,14,15]	Beta (10, 324)
Hepatobiliary toxicity	0.150 (0.120–0.180)	[9,14,15]	Beta (50, 284)
Infection	0.042 (0.034–0.050)	[9,14,15]	Beta (14, 320)
Vomiting	0.036 (0.029–0.043)	[9,14,15]	Beta (12, 322)
Abem + NSAI AEs incidence (Grade 3 or higher)			
Leukopenia	0.100 (0.080–0.120)	[16,17]	Beta (52, 480)
Anemia	0.080 (0.064–0.096)	[16,17]	Beta (42, 490)
Neutropenia	0.231 (0.185–0.277)	[16,17]	Beta (123, 409)
Thrombocytopenia	0.050 (0.040–0.060)	[16,17]	Beta (11, 521)
Nausea	0.008 (0.006–0.010)	[16,17]	Beta (4, 528)
Diarrhea	0.073 (0.059–0.088)	[16,17]	Beta (39, 493)
Fatigue	0.013 (0.010–0.016)	[16,17]	Beta (7, 525)
Hepatobiliary toxicity	0.077 (0.062–0.092)	[16,17]	Beta (41, 491)
Infection	0.030 (0.024–0.036)	[16,17]	Beta (16, 517)
Vomiting	0.017 (0.014–0.020)	[16,17]	Beta (9, 525)
Placebo + NSAI AEs incidence (Grade 3 or higher)			
Anemia	0.015 (0.012–0.018)	[6,9,11,12,13,14,15,16,17]	Beta (16, 1056)
Thrombocytopenia	0.004 (0.003–0.005)	[6,9,11,12,13,14,15,16,17]	Beta (4, 1068)
Neutropenia	0.015 (0.012–0.018)	[6,9,11,12,13,14,15,16,17]	Beta (16, 1056)
Leukopenia	0.006 (0.005–0.007)	[6,9,11,12,13,14,15,16,17]	Beta (7, 1065)
Nausea	0.008 (0.007–0.010)	[6,9,11,12,13,14,15,16,17]	Beta (8, 1064)
Diarrhea	0.007 (0.006–0.009)	[6,9,11,12,13,14,15,16,17]	Beta (8, 1064)
Fatigue	0.005 (0.004–0.006)	[6,9,11,12,13,14,15,16,17]	Beta (5, 1067)
Hepatobiliary toxicity	0.018 (0.015–0.022)	[6,9,11,12,13,14,15,16,17]	Beta (19, 1053)
Infection	0.018 (0.015–0.022)	[6,9,11,12,13,14,15,16,17]	Beta (19, 1053)
Vomiting	0.008 (0.007–0.010)	[6,9,11,12,13,14,15,16,17]	Beta (8, 1064)

Palbo + NSAI, palbociclib plus letrozole/anastrozole; Ribo + NSAI, ribociclib plus letrozole/anastrozole; Abem + NSAI, abemaciclib plus letrozole/anastrozole; placebo + NSAI, placebo plus letrozole/anastrozole; AEs, adverse events.

**Table 2:** Model parameters of costs and utilities: baseline values, ranges, and distributions.

Variable	Baseline Value (Range)	Reference	Distribution
Drug cost per dosage unit, $			
Palbociclib (125 mg)	30.54 (24.43–36.65)	Local database	Gamma (96.00, 3.14)
Ribociclib (200 mg)	42.00 (33.60–50.40)		Gamma (96.04, 2.29)
Abemaciclib (150 mg)	12.75 (10.20–15.30)		Gamma (96.04, 7.53)
Letrozole (2.5 mg)	0.32 (0.26–0.38)		Gamma (96.04, 300.13)
Anastrozole (1 mg)	0.42 (0.34–0.50)		Gamma (96.04, 228.67)
Supportive care	274 (219.20–328.80)	[22,23,24,25]	Gamma (96.04, 0.35)
Imaging/Surveillance	176.49 (141.19–211.79)	[22,23,24,25]	Gamma (96.03, 0.54)
Laboratory test	82.59 (66.07–99.11)	[22,23,24,25]	Gamma (96.02, 1.16)
End of life care	9032 (7225–10,838)	[22,23,24,25]	Gamma (96.04, 0.01)
AEs cost, $			
Anemia	6434 (5147.2–7720.8)	[22,23,24,25]	Gamma (96.04, 0.015)
Thrombocytopenia	3551 (2841.36–4262.04)	[22,23,24,25]	Gamma (96.04, 0.027)
Neutropenia	21,156 (16,924.8–25,387.2)	[22,23,24,25]	Gamma (96.04, 0.005)
Leukopenia	21,156 (16,924.8–25,387.2)	[22,23,24,25]	Gamma (96.04, 0.005)
Diarrhea	7377 (5901.6–8852.4)	[22,23,24,25]	Gamma (96.04, 0.013)
Hepatobiliary toxicity	7516 (6012.8–9019.2)	[22,23,24,25]	Gamma (96.04, 0.012)
Fatigue	6908 (5526.4–8289.6)	[22,23,24,25]	Gamma (96.04, 0.014)
Infection	10,128 (8102.4–12,153.6)	[22,23,24,25]	Gamma (96.04, 0.009)
Nausea	6182 (4945.6–7418.4)	[22,23,24,25]	Gamma (96.04, 0.016)
Vomiting	5246 (4196.8–6295.2)	[22,23,24,25]	Gamma (96.04, 0.018)
Pulmonary embolism	10,036 (8028.8–12,043.2)	[22,23,24,25]	Gamma (96.04, 0.010)
Discount rate, %	3	[22,23,24,25]	Beta (0.03, 0.97)
Body Weight (kg)	65 (32.5–97.5)	[22,23,24,25]	Gamma (15.37, 0.24)
Body surface area (m^2^)	1.72 (1.376–2.064)	[22,23,24,25]	Gamma (96.04, 55.84)
Utility			
Progression-free state (PR/CR)	0.8345 (0.6676–1.00)	[24,25]	Beta (0.8345, 0.1655)
Progression-free state (SD)	0.8296 (0.6637–0.9955)	[24,25]	Beta (0.8296, 0.1704)
Progression state (PD)	0.5050 (0.404–0.606)	[24,25]	Beta (0.505, 0.495)

**Table 3:** Baseline Results.

Strategy	Abem + NSAI	Palbo + NSAI	Ribo + NSAI	Placebo + NSAI
Cost, $				
Progression-free survival	40,164	48,202	109,132	8683
Overall	83,345	91,134	152,001	70,743
QALYs				
Progression-free survival	2.22	2.16	2.15	1.92
Overall	4.16	4.09	4.08	3.78
LYs	6.51	6.43	6.40	6.00
ICUR, $/QALY ^a^	33,163	65,777	270,860	—
ICER, $/LY ^a^	24,710	47,421	203,145	—
INHB, QALY ^a^	0.05	−0.23	−1.84	—
INMB, $ ^a^	1849	−8602	−69,849	—

^a^ Compared with placebo + NSAI group. NSAI, letrozole or anastrozole; Abem + NSAI, abemaciclib plus NSAI; Palbo + NSAI, palbociclib plus NSAI; Ribo + NSAI, ribociclib plus NSAI; placebo + NSAI, placebo plus NSAI; ICUR, incremental cost-utility ratio; ICER, incremental cost-effectiveness ratio; INHB, incremental net health benefit; INMB, incremental net monetary benefit; QALY, quality-adjusted life-year; LY, life year.

**Table 4:** Pairwise Comparisons of Baseline Results.

Groups		Vs. Abem + NSAI	vs. Palbo + NSAI	Vs. Ribo + NSAI	Vs. Placebo + NSAI
Abem + NSAI	ICUR, $/QALY	—	Dominate	Dominate	33,163
	INHB, QALY	—	0.27	1.89	0.05
	INMB, $	—	10,451	71,698	1849
Palbo + NSAI	ICUR, $/QALY	Dominated	—	Dominate	65,777
	INHB, QALY	−0.27	—	1.61	−0.23
	INMB, $	−10,451	—	61,247	−8602
Ribo + NSAI	ICUR, $/QALY	Dominated	Dominated	—	270,860
	INHB, QALY	−1.89	−1.61	—	−1.84
	INMB, $	−71,698	−61,247	—	−69,849
Placebo + NSAI	ICUR, $/QALY	Dominated	Dominated	Dominated	—
	INHB, QALY	−0.05	0.23	1.84	—
	INMB, $	−1849	8602	69,849	—

NSAI, letrozole or anastrozole; Abem + NSAI, abemaciclib plus NSAI; Palbo + NSAI, palbociclib plus NSAI; Ribo + NSAI, ribociclib plus NSAI; placebo + NSAI, placebo plus NSAI; ICUR, incremental cost-utility ratio; ICER, incremental cost-effectiveness ratio; INHB, incremental net health benefit; INMB, incremental net monetary benefit; QALY, quality-adjusted life-year.

## Data Availability

The data that support the findings of this study are available from the published studies, upon reasonable request.

## Supplementary Materials

The following supporting information can be downloaded at: https://www.mdpi.com/article/10.3390/cancers15133386/s1, Table S1: PRISMA NMA Checklist, Table S2: The CHEERS 2022 checklist, Table S3: Literature search strategies, Table S4: Characteristics of studies included in NMA, Table S5: Patient baseline demographic and clinical characteristics, Table S6: Summary of statistical goodness-of-fit of Kaplan-Meier curves of placebo + NSAI, Table S7: Background mortality rate in China, Table S8: Drug doses, schedule, and unit price, Figure S1: Kaplan-Meier curves fitting and extrapolation of placebo + NSAI, Figure S2: Literature search and selection, Figure S3: Comparative network plots for efficacy and toxicity of first-line treatment for postmenopausal women with HR+/HER2− advanced or metastatic breast cancer, Figure S4: Summary of quality assessments using Cochrane Risk of Bias Tool 2.0, Figure S5: Probabilities for each Markov states in every cycle, Figure S6. Probability sensitivity analysis scatter plot.
