# Supplementary material for: CDK4/6 Inhibitors in the First-Line Treatment of Postmenopausal Women with HR+/HER2− Advanced or Metastatic Breast Cancer: An Updated Network Meta-Analysis and Cost-Effectiveness Analysis
Source: Cancers (Basel). 2023 Jun 28;15(13):3386. doi: 10.3390/cancers15133386 (PMC10340287; doi:10.3390/cancers15133386)
Supplement: Supplementary file 1 [file cancers-15-03386-s001.zip › cancers-2388380-supplementary.pdf]

Supplement Tables and Figures

Supplementary Table S1. PRISMA NMA Checklist

Supplementary Table S2. The CHEERS 2022 checklist

Supplementary Table S3. Literature search strategies

Supplementary Table S4. Characteristics of studies included in NMA

Supplementary Table S5. Patient baseline demographic and clinical characteristics

Supplementary Table S6. Summary of statistical goodness-of-fit of Kaplan-Meier curves of placebo+ NSAI

Supplementary Table S7. Background mortality rate in China

Supplementary Table S8. Drug doses, schedule, and unit price

Supplementary Figure S1. Kaplan-Meier curves fitting and extrapolation of placebo+ NSAI

Supplementary Figure S2. Literature search and selection

Supplementary Figure S3. Comparative network plots for efficacy and toxicity of first-line treatment for postmenopausal women with HR+/HER2- advanced or metastatic breast cancer

Supplementary Figure S4. Summary of quality assessments using Cochrane Risk of Bias Tool 2.0

Supplementary Figure S5. Probabilities for each Markov states in every cycle

Supplementary Figure S6. Probability sensitivity analysis scatter plot

Supplementary Table S1. PRISMA NMA Checklist

**Section/Topic**	**Item #**	**Checklist Item**	**Reported on Page #**
**TITLE**			
Title	1	Identify the report as a systematic review *incorporating a network meta-analysis (or related form of meta-analysis).*	#1
**ABSTRACT**			
Structured summary	2	Provide a structured summary including, as applicable:<br>**Background:** main objectives<br>**Methods:** data sources; study eligibility criteria, participants, and interventions; study appraisal; and *synthesis methods, such as network meta-analysis.*<br>**Results:** number of studies and participants identified; summary estimates with corresponding confidence/credible intervals; *treatment rankings may also be discussed. Authors may choose to summarize pairwise comparisons against a chosen treatment included in their analyses for brevity.*<br>**Discussion/Conclusions:** limitations; conclusions and implications of findings.<br>**Other:** primary source of funding; systematic review registration number with registry name.	#2
**INTRODUCTION**			
Rationale	3	Describe the rationale for the review in the context of what is already known, *including mention of why a network meta- analysis has been conducted.*	#3-4
Objectives	4	Provide an explicit statement of questions being addressed, with reference to participants, interventions, comparisons, outcomes, and study design (PICOS).	#3-4
**METHODS**			
Protocol and registration	5	Indicate whether a review protocol exists and if and where it can be accessed (e.g., Web address); and, if available, provide registration information, including registration number.	#5
Eligibility criteria	6	Specify study characteristics (e.g., PICOS, length of follow-up) and report characteristics (e.g., years considered, language, publication status) used as criteria for eligibility, giving rationale.	#5

		Clearly describe eligible treatments included in the treatment network, and note whether any have been clustered or merged into the same node (with justification).	
Information sources	7	Describe all information sources (e.g., databases with dates of coverage, contact with study authors to identify additional studies) in the search and date last searched.	#5
Search	8	Present full electronic search strategy for at least one database, including any limits used, such that it could be repeated.	Supplementary Table S3
Study selection	9	State the process for selecting studies (i.e., screening, eligibility, included in systematic review, and, if applicable, included in the meta-analysis).	#5
Data collection process	10	Describe method of data extraction from reports (e.g., piloted forms, independently, in duplicate) and any processes for obtaining and confirming data from investigators	#5-6
Data items	11	List and define all variables for which data were sought (e.g., PICOS, funding sources) and any assumptions and simplifications made.	#5-6
**Geometry of the network**	**S1**	Describe methods used to explore the geometry of the treatment network under study and potential biases related to it. This should include how the evidence base has been graphically summarized for presentation, and what characteristics were compiled and used to describe the evidence base to readers	#6
Risk of bias within individual studies	12	Describe methods used for assessing risk of bias of individual studies (including specification of whether this was done at the study or outcome level), and how this information is to be used in any data synthesis.	#5
Summary measures	13	State the principal summary measures (e.g., risk ratio, difference in means). *Also describe the use of additional summary measures assessed, such as treatment rankings and surface under the cumulative ranking curve (SUCRA) values, as well as modified approaches used to present summary findings from meta-analyses.*	#5-6
Planned methods of analysis	14	Describe the methods of handling data and combining results of studies for each network meta-analysis. This	#5-6

		should include, but not be limited to:<br>• Handling of multi-arm trials;<br>• Selection of variance structure;<br>• Selection of prior distributions in Bayesian analyses; and<br>• Assessment of model fit.	
**Assessment of Inconsistency**	**S2**	Describe the statistical methods used to evaluate the agreement of direct and indirect evidence in the treatment network(s) studied. Describe efforts taken to address its presence when found.	#5-6
Risk of bias across studies	15	Specify any assessment of risk of bias that may affect the cumulative evidence (e.g., publication bias, selective reporting within studies).	#5-6
Additional analyses	16	Describe methods of additional analyses if done, indicating which were pre-specified. This may include, but not be limited to, the following:<br>• Sensitivity or subgroup analyses;<br>• Meta-regression analyses;<br>• Alternative formulations of the treatment network; and<br>• Use of alternative prior distributions for Bayesian analyses (if applicable).	N/A
**RESULTS†**			
Study selection	17	Give numbers of studies screened, assessed for eligibility, and included in the review, with reasons for exclusions at each stage, ideally with a flow diagram.	#9
**Presentation of network structure**	**S3**	Provide a network graph of the included studies to enable visualization of the geometry of the treatment network.	Supplement Figure S2
**Summary of network geometry**	**S4**	Provide a brief overview of characteristics of the treatment network. This may include commentary on the abundance of trials and randomized patients for the different interventions and pairwise comparisons in the network, gaps of evidence in the treatment network, and potential biases reflected by the network structure.	#9-10
Study characteristics	18	For each study, present characteristics for which data were extracted (e.g., study size, PICOS, follow-up period) and provide the citations.	Supplementary Table S4
Risk of bias within studies	19	Present data on risk of bias of each study and, if available, any outcome	#9-10

		level assessment.	
Results of individual studies	20	For all outcomes considered (benefits or harms), present, for each study: 1) simple summary data for each intervention group, and 2) effect estimates and confidence intervals. *Modified approaches may be needed to deal with information from larger networks.*	#9-10
Synthesis of results	21	Present results of each meta-analysis done, including confidence/credible intervals. *In larger networks, authors may focus on comparisons versus a particular comparator (e.g. placebo or standard care), with full findings presented in an appendix. League tables and forest plots may be considered to summarize pairwise comparisons.* If additional summary measures were explored (such as treatment rankings), these should also be presented.	Figure 2
**Exploration for inconsistency**	**S5**	Describe results from investigations of inconsistency. This may include such information as measures of model fit to compare consistency and inconsistency models, *P* values from statistical tests, or summary of inconsistency estimates from different parts of the treatment network.	N/A
Risk of bias across studies	22	Present results of any assessment of risk of bias across studies for the evidence base being studied.	Supplement Figure S4
Results of additional analyses	23	Give results of additional analyses, if done (e.g., sensitivity or subgroup analyses, meta-regression analyses, *alternative network geometries studied, alternative choice of prior distributions for Bayesian analyses,* and so forth).	N/A
**DISCUSSION**			
Summary of evidence	24	Summarize the main findings, including the strength of evidence for each main outcome; consider their relevance to key groups (e.g., healthcare providers, users, and policy-makers).	#12-13
Limitations	25	Discuss limitations at study and outcome level (e.g., risk of bias), and at review level (e.g., incomplete retrieval of identified research, reporting bias). *Comment on the validity of the assumptions, such as transitivity and consistency. Comment*	#14

		on any concerns regarding network geometry (e.g., avoidance of certain comparisons).	
Conclusions	26	Provide a general interpretation of the results in the context of other evidence, and implications for future research.	#15
**FUNDING**			
Funding	27	Describe sources of funding for the systematic review and other support (e.g., supply of data); role of funders for the systematic review. This should also include information regarding whether funding has been received from manufacturers of treatments in the network and/or whether some of the authors are content experts with professional conflicts of interest that could affect use of treatments in the network.	NA

PICOS = population, intervention, comparators, outcomes, study design.

* Text in italics indicate S wording specific to reporting of network meta-analyses that has been added to guidance from the PRISMA statement.

† Authors may wish to plan for use of appendices to present all relevant information in full detail for items in this section.

Supplementary Table S2. The CHEERS 2022 checklist

Section/item	Item No	Recommendation	Reported in section
**Title and abstract**			
Title	1	Identify the study as an economic evaluation and specify the interventions being compared.	Page 1
Abstract	2	Provide a structured summary that highlights context, key methods, results, and alternative analyses.	Page 2
**Introduction**			
Background and objectives	3	Give the context for the study, the study question, and its practical relevance for decision making in policy or practice.	Page 3-4
**Methods**			
Health economic analysis plan	4	Indicate whether a health economic analysis plan was developed and where available.	Page 6
Study population	5	Describe characteristics of the study population (such as age range, demographics, socioeconomic, or clinical characteristics).	Page 7
Setting and location	6	Provide relevant contextual information that may influence	Page 6-7
Comparators	7	Describe the interventions or strategies being compared and why chosen.	Page 6-7
Perspective	8	State the perspective(s) adopted by the study and why chosen.	Page 6
Time horizon	9	State the time horizon for the study and why appropriate.	Page 8
Discount rate	10	Report the discount rate(s) and reason chosen.	Page 8
Selection of outcomes	11	Describe what outcomes were used as the measure(s) of benefit(s) and harm(s).	Page 6
Measurement of outcomes	12	Describe how outcomes used to capture benefit(s) and harm(s)	Page 7-8
Valuation of outcomes	13	Describe the population and methods used to measure and value outcomes.	Page 7-8
Measurement and valuation of resources and costs	14	Describe how costs were valued.	Page 8-9
Currency, price date, and conversion	15	Report the dates of the estimated resource quantities and unit costs, plus the currency and year of conversion.	Page 8-9
Rationale and description of model	16	If modelling is used, describe in detail and why used. Report if the model is publicly available and where it can be accessed.	Page 7-8
Analytics and assumptions	17	Describe any methods for analysing or statistically transforming data, any extrapolation methods, and approaches for validating any model used.	Page 8-9

Characterizing heterogeneity	18	Describe any methods used for estimating how the results of the study vary for subgroups.	N/A
Characterizing distributional effects	19	Describe how impacts are distributed across different individuals or adjustments made to reflect priority populations.	Page 8-9
Characterizing uncertainty	20	Describe methods to characterise any sources of uncertainty in the analysis.	N/A
Approach to engagement with patients and others affected by the	21	Describe any approaches to engage patients or service recipients, the general public, communities, or stakeholders (such as clinicians or payers) in the design of the study.	Page 9
**Results**			
Study parameters	22	Report all analytic inputs (such as values, ranges, references) including uncertainty or distributional assumptions.	Page 10
Summary of main results	23	Report the mean values for the main categories of costs and outcomes of interest and summarise them in the most appropriate overall measure.	Page 10
Effect of uncertainty	24	Describe how uncertainty about analytic judgments, inputs, or projections affect findings. Report the effect of choice of discount rate and time horizon, if applicable.	Page 11
Effect of engagement with patients and others affected by the study	25	Report on any difference patient/service recipient, general public, community, or stakeholder involvement made to the approach or findings of the study	Page 11
**Discussion**			
Study findings, limitations, generalizability, and current knowledge	26	Report key findings, limitations, ethical or equity considerations not captured, and how these could affect patients, policy, or practice.	Page 12-13
**Other relevant information**			
Source of funding	23	Describe how the study was funded and any role of the funder in the identification, design, conduct, and reporting of the analysis	Page 15
Conflicts of interest	28	Report authors conflicts of interest according to journal or International Committee of Medical Journal Editors requirements.	Page 15

Supplementary Table S3. Literature search strategies

Database	Medical subject headings (MeSH) and Keywords
**PubMed**	
**#1**	"palbociclib"[Title/Abstract] OR "Ibrance"[Title/Abstract] OR "ribociclib"[Title/Abstract] OR "LEE011" [Title/Abstract] OR "Kisqali"[Title/Abstract] OR "abemaciclib"[Title/Abstract] OR "Verzenio"[Title/Abstract] OR "dalpiciclib"[Title/Abstract] OR "CDK4/6 inhibitors"[Title/Abstract]
**#2**	"letrozole"[Title/Abstract] OR "Femara"[Title/Abstract] OR "anastrozole"[Title/Abstract] OR "anastrazole" [Title/Abstract] OR "arimidex"[Title/Abstract] OR "exemestane"[Title/Abstract] OR "examestane"[Title/Abstract] OR "aromasil"[Title/Abstract] OR "aromasin"[Title/Abstract] OR "aromasine"[Title/Abstract] OR "Aromatase Inhibitors"[Title/Abstract]
**#3**	"Breast Neoplasm"[MeSH Terms] OR"Neoplasm, Breast" [Title/Abstract] OR "Breast Tumors"[Title/Abstract]OR"Tumor, Breast"[Title/Abstract] OR "Breast Cancer"[Title/Abstract]OR"Mammary Cancer"[Title/Abstract] OR "Malignant Neoplasm of Breast"[Title/Abstract]OR "Breast Malignant Neoplasm"[Title/Abstract]OR"Malignant Tumor of Breast"[Title/Abstract] OR "Breast Malignant Tumor"[Title/Abstract]OR"Cancer of Breast"[Title/Abstract] OR "Human Mammary Carcinomas"[Title/Abstract]OR"Human Mammary Neoplasm"[Title/Abstract]OR"Breast Carcinoma"[Title/Abstract]
**#4**	"clinical trials as topic"[MeSH Terms] OR "clinical trial"[Publication Type] OR "phase III"[Title/Abstract] OR "phase 3"[Title/Abstract] OR "clinical trials"[Title/Abstract]"phase II"[Title/Abstract] OR "phase 2"[Title/Abstract] OR "real world"[Title/Abstract] OR "real-world" [Title/Abstract] OR "real life"[Title/Abstract] OR"real-life"[Title/Abstract]
**#5**	("2015/01/01"[Date - Publication] : "2023/02/10"[Date - Publication])
**#6**	#1 AND #2 AND #3 AND #4 AND #5
**#7**	"review"[Article type] OR "meta"[Title] OR "meta-analysis"[Title] OR "protocol"[Title]
**#8**	#6 NOT #7
**EMBASE**	
**1**	(palbociclib OR Ibrance OR ribociclib OR LEE011 OR Kisqali OR abemaciclib OR Verzenio OR dalpiciclib OR 'CDK4/6 inhibitors'):ti,ab,kw
**2**	(letrozole OR Femara OR anastrozole OR anastrazole OR arimidex OR exemestane OR examestane OR aromasil OR aromasin OR aromasine OR 'aomatase Inhibitors'):ti,ab,kw
**3**	('Breast Neoplasm' OR 'Neoplasm, Breast' OR 'Breast Tumors' OR 'Tumor, Breast' OR 'Breast Cancer' OR 'Mammary Cancer' OR 'Malignant Neoplasm of Breast' OR 'Breast Malignant Neoplasm' OR 'Malignant Tumor of Breast' OR 'Breast Malignant Tumor' OR 'Cancer of Breast' OR 'Human Mammary Carcinomas' OR 'Human Mammary Neoplasm' OR 'Breast Carcinoma'):ti,ab,kw
**4**	'clinical trials' OR 'phase 3 clinical trial' OR 'phase III clinical trial' OR 'phase II clinical trial' OR 'phase 2 clinical trial' OR 'real world ' OR 'real- world ' OR 'real life' OR 'real-life'

**5**	[1-1-2015]/sd NOT [10-02-2023]/sd
**6**	1 AND 2 AND 3 AND 4AND 5
**Web of Science**	
**1**	TS = (palbociclib OR ribociclib OR abemaciclib OR dalpiciclib OR 'CDK4/6 inhibitors')
**2**	TS=(letrozole OR anastrozole OR anastrazole OR exemestane OR examestane OR aromasil OR aromasin OR aromasine OR 'aomatase Inhibitors')
**3**	TS = ('Breast Neoplasm' OR 'Neoplasm, Breast' OR 'Breast Tumors' OR 'Tumor, Breast' OR 'Breast Cancer' OR 'Mammary Cancer' OR 'Malignant Neoplasm of Breast' OR 'Breast Malignant Neoplasm' OR 'Malignant Tumor of Breast' OR 'Breast Malignant Tumor' OR 'Cancer of Breast' OR 'Human Mammary Carcinomas' OR 'Human Mammary Neoplasm' OR 'Breast Carcinoma')
**4**	TS = ('clinical trials' OR 'phase 3 clinical trial' OR 'phase III clinical trial' OR 'phase II clinical trial' OR 'phase 2 clinical trial' OR 'real world ' OR 'real- world ' OR 'real life' OR 'real-life')
**5**	PY = ('2015-2023)
**6**	1 AND 2 AND 3 AND 4AND 5
**Cochrane Library**	
**1**	(palbociclib OR Ibrance OR ribociclib OR LEE011 OR Kisqali OR abemaciclib OR Verzenio OR dalpiciclib OR 'CDK4/6 inhibitors')
**2**	(letrozole OR Femara OR anastrozole OR anastrazole OR arimidex OR exemestane OR examestane OR aromasil OR aromasin OR aromasine OR 'aomatase Inhibitors')
**3**	('Breast Neoplasm' OR 'Neoplasm, Breast' OR 'Breast Tumors' OR 'Tumor, Breast' OR 'Breast Cancer' OR 'Mammary Cancer' OR 'Malignant Neoplasm of Breast' OR 'Breast Malignant Neoplasm' OR 'Malignant Tumor of Breast' OR 'Breast Malignant Tumor' OR 'Cancer of Breast' OR 'Human Mammary Carcinomas' OR 'Human Mammary Neoplasm' OR 'Breast Carcinoma')
**4**	('clinical trials' OR 'phase 3 clinical trial' OR 'phase III clinical trial')
**5**	Publication date: Between Jan 2015 and Feb 2023
**6**	1 AND 2 AND 3 AND 4 AND 5
**ClinicalTrials.gov**	
**1**	Condition or disease: palbociclib OR Ibrance OR ribociclib OR LEE011 OR Kisqali OR abemaciclib OR Verzenio OR dalpiciclib OR 'CDK4/6 inhibitors'
**2**	Condition or disease: letrozole OR Femara OR anastrozole OR anastrazole OR arimidex OR exemestane OR examestane OR aromasil OR aromasin OR aromasine OR 'aomatase Inhibitors'
**3**	Condition or disease: 'Breast Neoplasm' OR 'Neoplasm, Breast' OR 'Breast Tumors' OR 'Tumor, Breast' OR 'Breast Cancer' OR 'Mammary Cancer' OR 'Malignant Neoplasm of Breast' OR 'Breast Malignant Neoplasm' OR 'Malignant Tumor of Breast' OR 'Breast Malignant Tumor' OR 'Cancer of Breast' OR 'Human Mammary Carcinomas' OR 'Human Mammary Neoplasm' OR 'Breast Carcinoma'
**4**	Phase: phase 3
**5**	1 AND 2 AND 3 AND 4

Supplementary Table S4. Characteristics of RCTs included in NMA

Trial name	Source	Registered ID (Randomization)	Total sample size	Intervention Arm	Control Arm	Median OS (months)	HR for OS (95% CI)	Median PFS (months)	HR for PFS (95% CI)
**PALOMA-1**	Lancet. Oncology and Breast cancer research and treatment	NCT00721409	165	Palbociclib plus Letrozole	Letrozole alone	37.5 vs 34.5	0.897 (0.623-1.294)	20.20 vs 10.20	0.488 (0.319-0.748)
**PALOMA-2**	2022 ASCO and Breast cancer research and treatment	NCT01740427	666	Palbociclib plus Letrozole	Placebo plus Letrozole	51.6vs 44.6	0.869 (0.706-1.069)	27.60 vs 14.50	0.563 (0.461-0.687)
**PALOMA-4**	European journal of cancer	NCT02297438	340	Palbociclib plus Letrozole	Placebo plus Letrozole	NR vs NR	NR	21.50 vs 13.90	0.680 (0.530-0.870)
**P-reality X**	NPJ breast cancer	NCT05361655	2888	Palbociclib plus Letrozole/Anastrozole	Letrozole/Anastrozole alone	49.1 vs 43.2	0.760 (0.650-0.870)	19.30 vs 13.90	0.700 (0.620-0.780)

**MONALEESA-2**	Ann Oncol and The New England journal of medicine	NCT01958021	668	Ribociclib plus Letrozole	Placebo plus Letrozole	63.9 vs 51.4	0.760 (0.630-0.930)	25.30 vs 16.0	0.568 (0.457-0.704)
**MONARCH-3**	NPJ breast cancer and 2022 ESMO	NCT02246621	493	Abemaciclib plus Letrozole/Anastrozole	Placebo plus Letrozole/Anastrozole	67.1 vs 54.5	0.754 (0.584-0.974)	28.18 vs 14.76	0.540 (0.418-0.698)
**MONARCH plus**	2022 CSCO	NCT02763566	207	Abemaciclib plus Letrozole/Anastrozole	Placebo plus Letrozole/Anastrozole	NR vs NR	NR	28.30 vs 14.70	0.476 (0.348-0.649)

ASCO, American Society of Clinical Oncology; ESMO, European Society of Medical Oncology; CSCO, Chinese Society of Clinical Oncology; OS, overall survival; PFS, progression-free survival; HR, hazard ratio; CI, confidence interval; NR, not reached.

Supplementary Table S5. Patient baseline demographic and clinical characteristics

Characteristics	Palbociclib								Ribociclib		Abemaciclib			
	PALOMA-1		PALOMA-2		PALOMA-4		P-reality X		MONALEESA-2		MORNARCH-3		MORNACH plus(cohortA)	
	Palbo +LET	LET alone	Palbo +LET	PBO+ LET	Palbo +LET	PBO+ LET	Palbo + NSAI	NSAI alone	Ribo +LET	PBO+ LET	Abem + NSAI	PBO+ NSAI	Abem + NSAI	PBO+ NSAI
**Sample size**	84	81	444	222	169	171	1572	1137	334	334	328	165	207	99
**Age – years**														
Median age	63	64	62	61	54	54	70	70	62	63	63	63	54	54
Range	54-71	56-70	30-89	28-88	31-70	29-70	63-78	63-79	23–91	29-88	38-87	32-88	32-83	27-77
**Ethnicity — no. (%)**														
Asian	NR	NR	65 (14.6)	30 (13.5)	169 (100)	171 (100)	0 (0)	0 (0)	28 (8.4)	23 (6.9)	103 (31.4)	45 (27.3)	182 (87.9)	89 (89.9)
Other	NR	NR	379 (85.4)	192 (86.5)	0 (0)	0 (0)	100 (100)	100 (100)	306 (91.6)	311 (93.1)	197 (60.1)	109 (66.0)	25 (12.1)	10 (10.1)
**ECOG performance score — no. (%)**														
0	46 (55)	45 (56)	257 (57.9)	102 (45.9)	84 (49.7)	81 (47.4)	472 (30.1)	348 (30.6)	205 (61.4)	202 (60.5)	192 (58.5)	104 (63.0)	NR	NR
1	38 (45)	36 (44)	178 (40.1)	117 (52.7)	85 (50.3)	90 (52.6)	362 (23.0)	259 (22.8)	129 (38.6)	132 (39.5)	136 (41.5)	61 (37.0)	NR	NR
**disease setting, no. (%)**														
De novo metastatic	NR	NR	139 (31.3)	71 (32.0)	NR	NR	NR	NR	NR	NR	135 (41.2)	61 (37.0)	41 (19.8)	22 (22.2)
metastatic recurrent	NR	NR	294 (66.2)	145 (65.3)	NR	NR	NR	NR	NR	NR	182 (55.5)	99 (60.0)	157 (75.8)	70 (70.7)
locoregionally recurrent	NR	NR	11 (2.6)	6 (2.8)	NR	NR	NR	NR	NR	NR	11 (3.4)	5 (3.0)	8 (3.9)	7 (7.1)
**metastatic site no. (%)**														

visceral	37 (44)	43 (53)	214 (48.2)	110 (49.5)	94 (55.6)	96 (56.1)	460 (29.3)	337 (29.7)	197 (59.0)	196 (58.7)	172 (52.4)	89 (53.9)	126 (60.9)	59 (59.6)
bone only	17 (20)	12 (15)	103 (23.2)	48 (21.6)	NR	NR	589 (37.5)	440 (38.7)	69 (20.7)	78 (23.4)	70 (21.3)	39 (21.8)	NR	NR
other	30 (36)	26 (32)	127 (28.6)	64 (28.9)	75 (44.4)	75 (43.9)	523 (33.2)	360 (31,6)	NR	NR	86 (26.2)	37 (22.4)	81 (39.1)	40 (40.4)
**no. of metastatic site-no. (%)**														
<3	NR	NR	255 (57.5)	118 (53.1)	87 (51.4)	88 (51.5)	1145 (72.8)	850 (74.7)	218 (64.9)	220 (65.8)	172 (53.0)	89 (54.5)	NR	NR
≥3	NR	NR	189 (42.5)	104 (46.9)	82 (48.6)	83 (48.5)	242 (15.5)	176 (15.4)	114 (34.1)	113 (33.8)	154 (47.0)	75 (45.5)	NR	NR
**previous treatment -no. (%)**														
Adjuvant chemotherapy	34 (40.0)	37 (46.0)	213 (48.0)	109 (49.1)	126 (74.6)	129 (75.4)	NR	NR	146 (43.7)	145 (43.4)	125 (38.1)	66 (40.0)	140 (67.6)	63 (63.6)
Adjuvant endocrine therapy	27 (32.0)	28 (35.0)	249 (56.1)	126 (56.8)	102 (60.4)	109 (63.7)	NR	NR	175 (52.4)	171 (51.2)	150 (45.7)	80 (48.5)	121 (58.5)	61 (61.6)
Aromatase inhibitor	14 (17.0)	14 (17.0)	122 (27.5)	58 (26.2)	27 (16.0)	22 (12.9)	NR	NR	100 (30.0)	92 (27.5)	85 (25.9)	50 (30.3)	20 (9.7)	13 (13.1)
other endocrine therapy	24 (29.0)	24 (30.0)	224 (50.5)	109 (49.1)	101 (59.8)	101 (59.1)	NR	NR	146 (43.7)	152 (45.5)	65 (19.8)	30 (18.2)	101 (48.8)	48 (48.5)

The baseline demographic and clinical characteristics of the hypothetical patients in the model were derived from the relevant data of PALOMA-1、PALOMA-2、PALOMA-4 and P-reality X、MONALEESA-2、MORNARCH-3 and MORNACH plus trials.

ECOG, Eastern Cooperative Oncology Group; Palbo, palbociclib, Ribo, ribociclib, Abem, abemaciclib, LET, letrozole, NSAI, letrozole/anastrozole, PBO, placebo.

Supplementary Table S6. Statistical goodness-of-fit of Kaplan-Meier curves of Placebo+NSAI based on PALOMA-1 trial.

	**Placebo+NSAI PFS curve**		**Placebo +NSAI OS curve**	
	**AIC**	**BIC**	**AIC**	**BIC**
**Exponential**	-236.8787	-234.8017	-322.5642	-317.2563
**Weibull**	-238.6653	-231.0832	-400.2639	-392.302
**Log-logistic**	-188.1303	-182.3348	-527.1055	-519.1436
**Lognormal**	-191.4921	-185.6966	-525.3918	-517.4299
**Gompertz**	-196.291	-190.4955	-406.6738	-398.7119

OS, overall survival; PFS, progression-free survival; AIC, Akaike's information criterion; BIC, Bayesian information criterion; NSAI, letrozole/anastrozole.

Supplementary Table S7. Background mortality rate in China

Age	Background mortality rate	Age	Background mortality rate	Age	Background mortality rate
23	0.00054	50	0.00363	77	0.04970
24	0.00056	51	0.00374	78	0.05466
25	0.00058	52	0.00397	79	0.06025
26	0.00057	53	0.00440	80	0.07162
27	0.00059	54	0.00497	81	0.07499
28	0.00061	55	0.00517	82	0.08228
29	0.00068	56	0.00562	83	0.08934
30	0.00070	57	0.00607	84	0.09852
31	0.00077	58	0.00679	85	0.10516
32	0.00081	59	0.00764	86	0.11221
33	0.00083	60	0.00850	87	0.12213
34	0.00094	61	0.00934	88	0.13448
35	0.00103	62	0.01033	89	0.14546
36	0.00106	63	0.01106	90	0.16220
37	0.00114	64	0.01293	91	0.16954
38	0.00121	65	0.01411	92	0.18356
39	0.00134	66	0.01463	93	0.18787
40	0.00151	67	0.01708	94	0.18915
41	0.00155	68	0.01847	95	0.19821
42	0.00182	69	0.02167	96	0.19900
43	0.00189	70	0.02525	97	0.18581
44	0.00207	71	0.02638	98	0.18027
45	0.00231	72	0.03047	99	0.22825
46	0.00236	73	0.03304	100	0.66667
47	0.00254	74	0.03675		
48	0.00311	75	0.04067		
49	0.00327	76	0.04132		

The background mortality rate in the model was estimated based on the China life table. The start age was assumed as 61 years old, which was based on the median age in the baseline characteristic.

Supplementary Table S8. Drug doses, schedule, and unit price

Drug	Dose	Unit price ($)	Cost for one cycle ($/4wks)	Reference
**Progression free disease state**				
Palbociclib	125 mg PO QD 3-weeks-on, 1-week-off, each 4 weeks cycle	30.54/125mg	641.34	Local database
Ribociclib	600 mg PO QD 3-weeks-on, 1-week-off, each 4 weeks cycle	42.00/200mg	2646.00	Local database
Abemaciclib	150 mg PO BID on a continuous schedule	12.75/150mg	714.00	Local database
Letrozole	2.5 mg PO QD on a continuous schedule	0.32/2.5mg	9.00	Local database
Anastrozole	1.0 mg PO QD on a continuous schedule	0.42/1mg	11.89	Local database
**Progression disease state**				
Second-line therapy proportion in Palbo+NSAI, Ribo+NSAI, Abem+NSAI groups				
Exemestane	25 mg PO QD on a continuous schedule	0.75/25mg	21.13	Local database
Letrozole	2.5 mg PO QD on a continuous	0.32/2.5mg	9.00	Local database
Anastrozole	1.0 mg PO QD on a continuous schedule	0.42/1mg	11.89	Local database
Fulvestrant	500 mg IV each 4 weeks cycle	294/250mg	588	Local database
Tamoxifen	10 mg PO BID on a continuous schedule	0.18/10mg	10.08	Local database
Everolimus	10 mg PO QD on a continuous schedule	22.2/5mg	1243.2	Local database
Anthracyclines	90 mg/m² IV on day 1 each 3 weeks cycle	12.98/10mg	267.87	Local database
Capecitabine	1000 mg/m² PO bid 2-weeks-on, 1-week-off, each 3 weeks cycle	0.28/500mg	35.58	Local database
Gemcitabine	1000 mg/m² IV on days 1 and 8 each 3 weeks cycle	8.87/200mg	212.88	Local database
Docetaxel	75 mg/m² IV on day 1 each 3 weeks cycle	43.96/20mg	378.05	Local database
Vinorelbine	60 mg/m² IV on days 1 ,8 and 15 every 4 weeks cycle	18/10mg	557.28	Local database
Chidamide	30 mg PO BIW	51.45/5mg	2469.6	Local database
Second-line therapy proportion in PBO+NSAI groups				
Exemestane	1.0 mg PO QD on a continuous schedule	0.75/25mg	21.13	Local database

Letrozole	2.5 mg PO QD on a continuous	0.32/2.5mg	9.00	Local database
Anastrozole	1.0 mg PO QD on a continuous schedule	0.42/1mg	11.89	Local database
Fulvestrant	500 mg IV each 4 weeks cycle	294/250mg	588	Local database
Tamoxifen	10 mg PO BID on a continuous schedule	0.18/10mg	10.08	Local database
Everolimus	10 mg PO QD on a continuous schedule	22.2/5mg	1243.2	Local database
Anthracyclines	90 mg/m² IV on day 1 each 3 weeks cycle	12.98/10mg	267.87	Local database
Capecitabine	1000 mg/m² PO bid 2-weeks-on, 1-week-off, each 3 weeks cycle	0.28/500mg	35.58	Local database
Gemcitabine	1000 mg/m² IV on days 1 and 8 each 3 weeks cycle	8.87/200mg	212.88	Local database
Docetaxel	75 mg/m² IV on day 1 each 3 weeks cycle	43.96/20mg	378.05	Local database
Vinorelbine	60 mg/m² IV on days 1 ,8 and 15 every 4 weeks cycle	18/10mg	557.28	Local database
Chidamide	30 mg PO BIW	51.45/5mg	2469.6	Local database
Palbociclib	125 mg PO QD 3-weeks-on, 1-week-off, in 28-day treatment cycles	30.54/125mg	641.34	Local database
Ribociclib	600 mg PO QD 3-weeks-on, 1-week-off, in 28-day treatment cycles	42.00/200mg	2646.00	Local database
Abemaciclib	150 mg PO BID on a continuous schedule each 4 weeks cycle	12.75/150mg	714.00	Local database
Dalpiciclib	150 mg PO QD 3-weeks-on, 1-week-off, in 28-day treatment cycles	30.75/150mg	645.75	Local database

Palbo, palbociclib, Ribo, ribociclib, Abem, abemaciclib, LET, letrozole, NSAI, letrozole/anastrozole, PBO, placebo.

Supplementary Figure S1. Kaplan-Meier curves fitting and extrapolation of placebo+NSAI

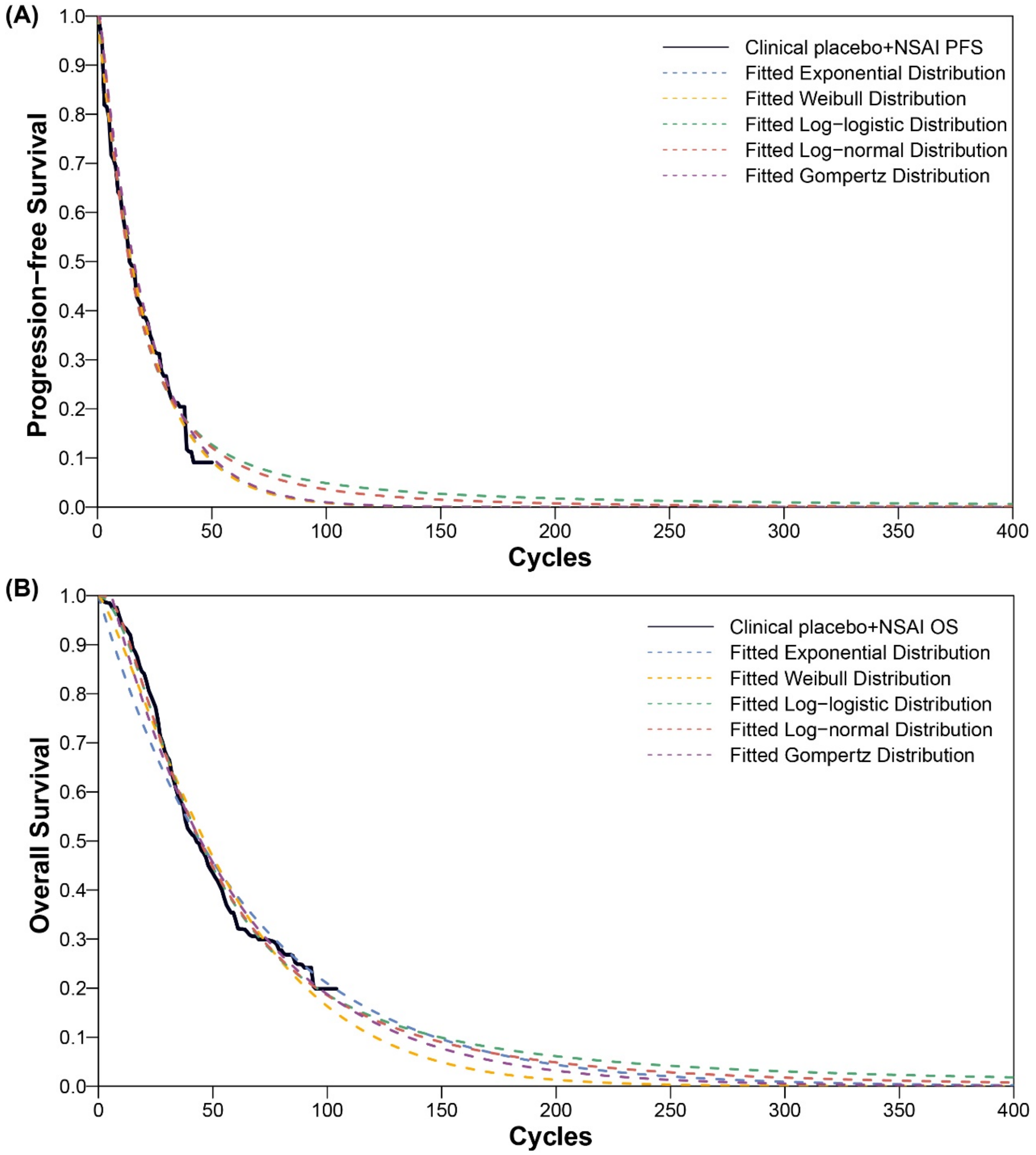

(A) Fitting and extrapolation of the progression-free survival curves; (B) Fitting and extrapolation of the overall survival curves.

NSAI, letrozole/anastrozole, PFS, progression-free survival; OS, overall survival.

Supplementary Figure S2. Literature search and selection.

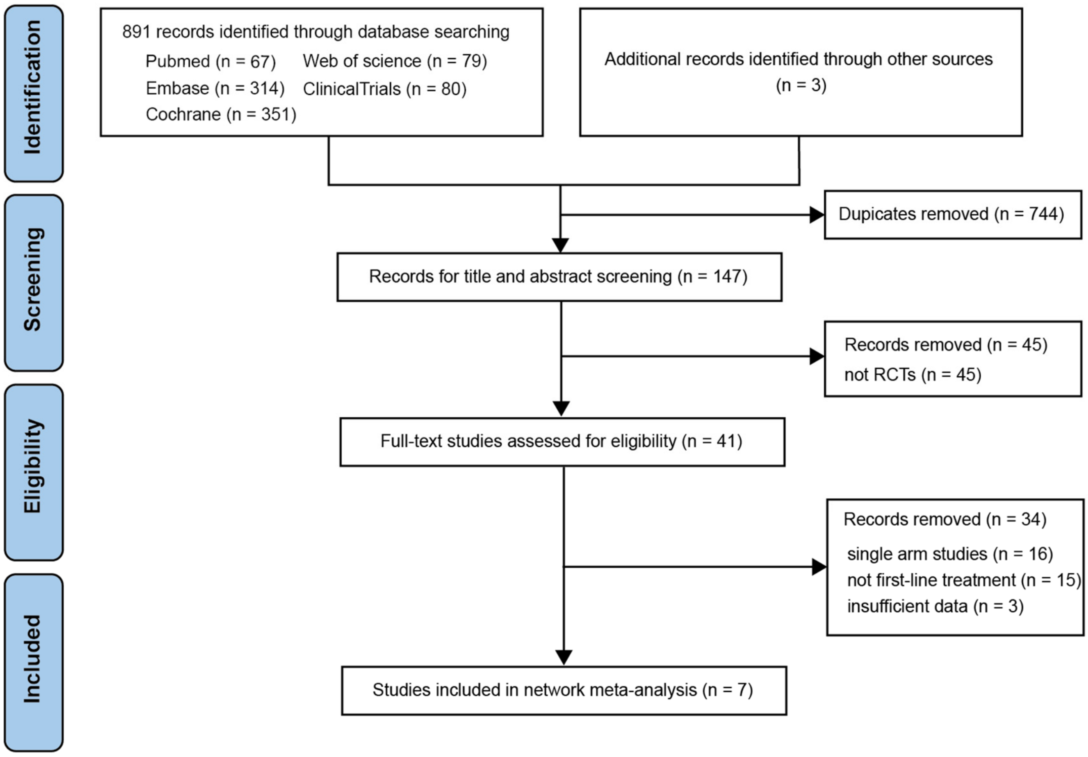

The study process followed the PRISMA guidelines. RCTs, randomized clinical trials.

Supplementary Figure S3. Comparative network plots for efficacy and toxicity of first-line treatment for patients with HR+/HER2- Advanced or Metastatic Breast Cancer

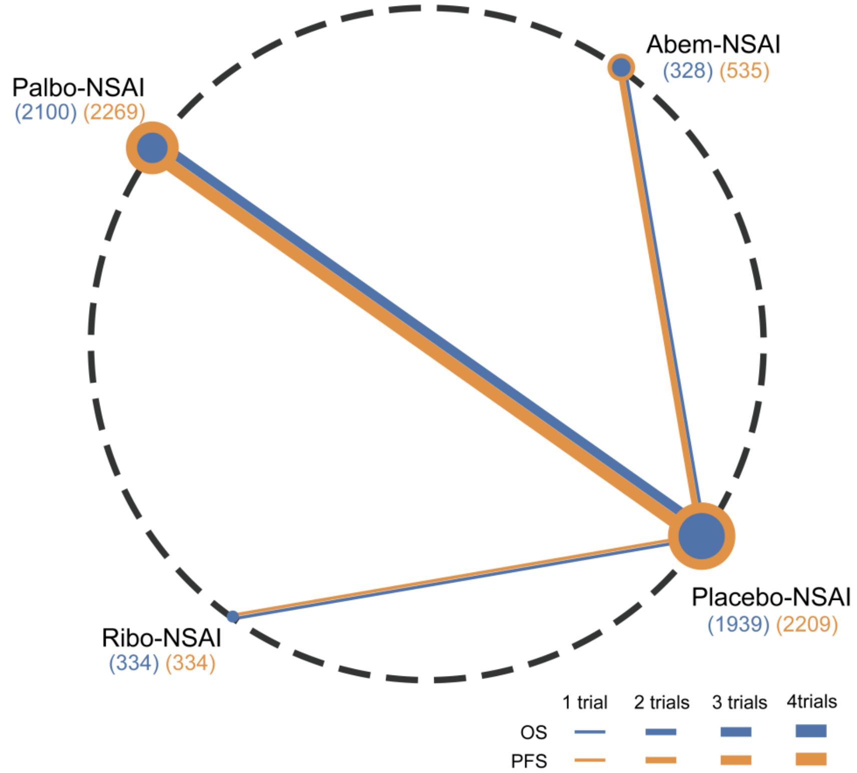

Comparisons were generated by using the Bayesian framework on OS, PFS. Each node (circle) represents a competing treatment in the network. The size of the nodes and the width of the lines are proportional to the number of randomized controlled trials and comparisons, respectively.

Palbo, palbociclib, Ribo, ribociclib, Abem, abemaciclib, NSAI, letrozole/anastrozole, PBO, placebo; PFS, progression-free survival; OS, overall survival.

Supplementary Figure S4. Summary of quality assessments using Cochrane Risk of Bias Tool 2.0

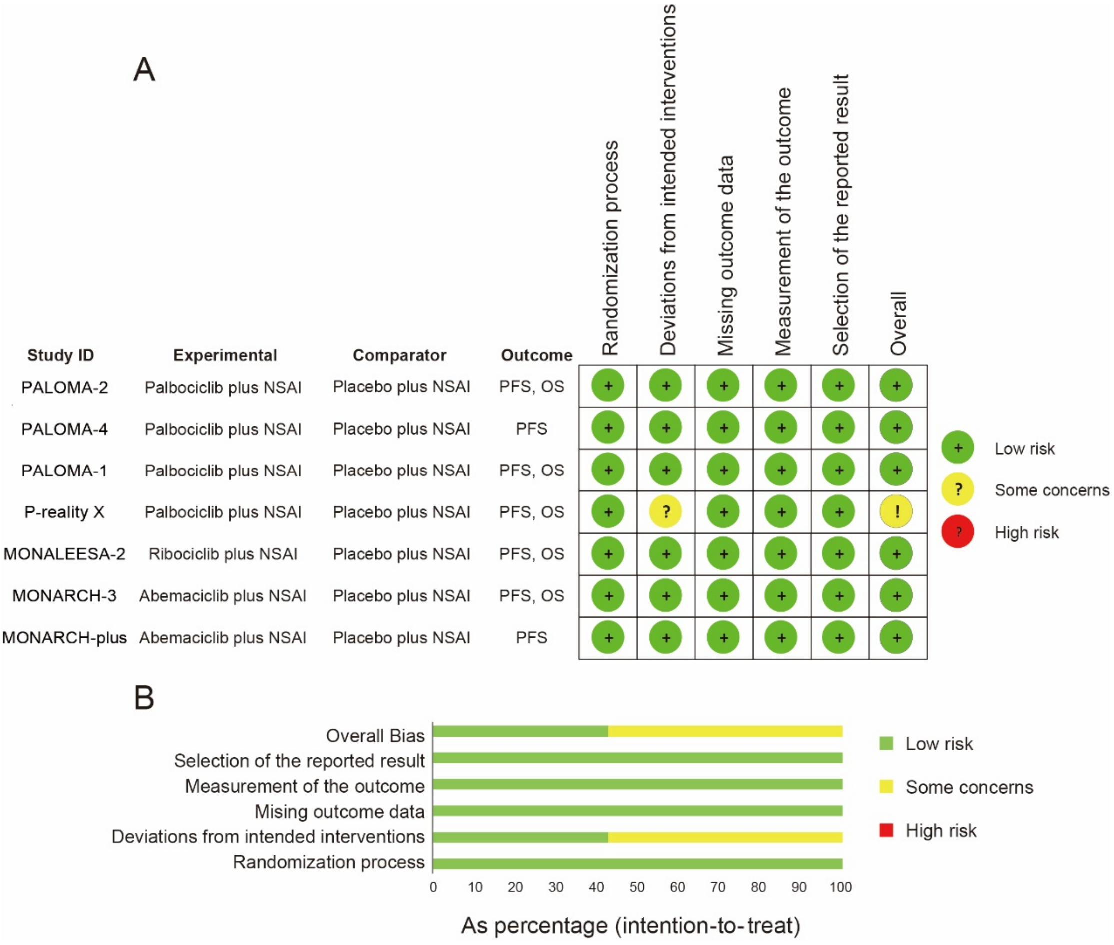

Studies were classified into one of three categories: low, high risk, or having “some concerns”.

Supplementary Figure S5. Probabilities for each Markov states in every cycle

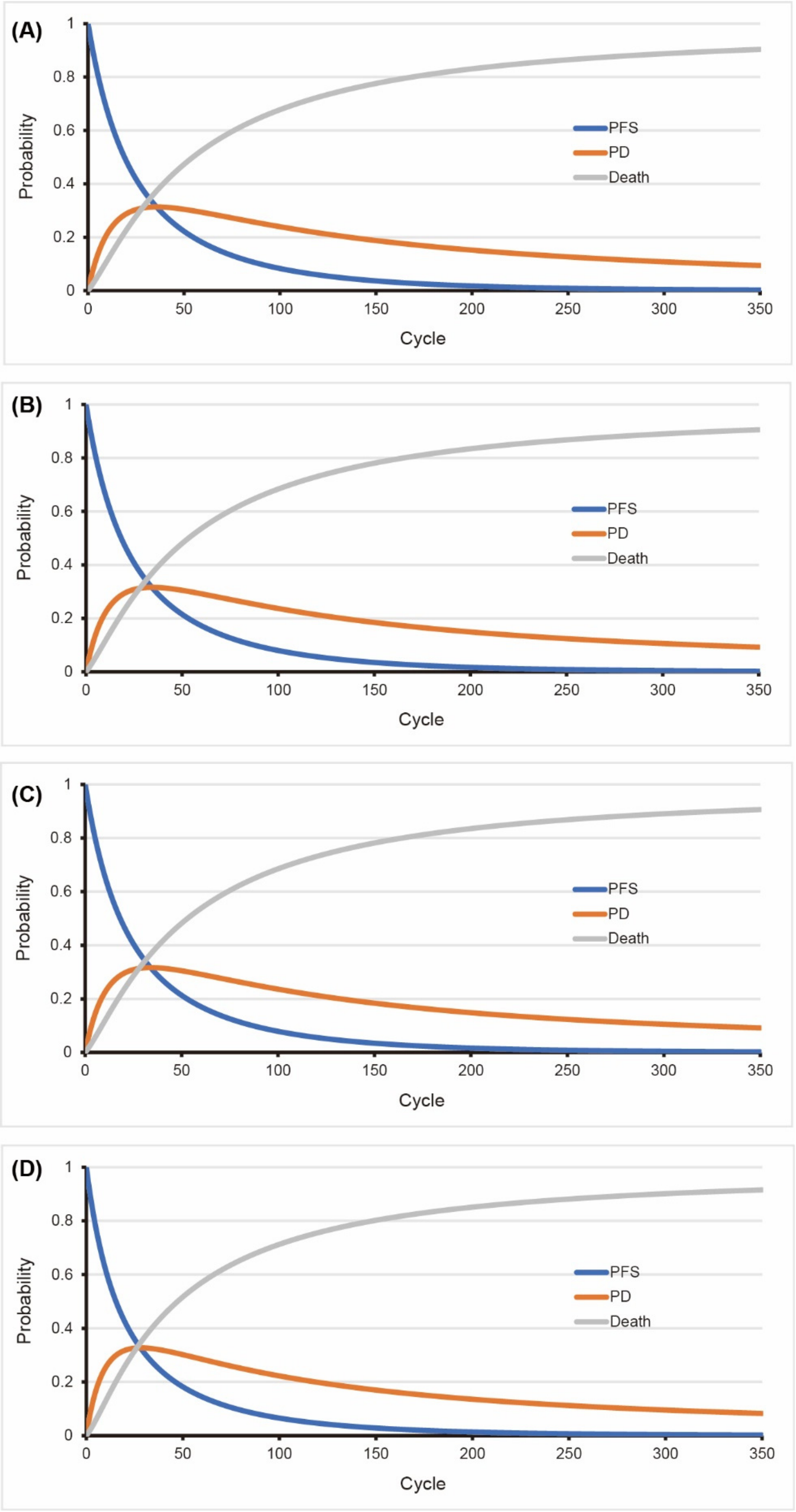

(A) Abemaciclib plus NSAI group; (B) Palbociclib plus NSAI group; (C) Ribociclib plus NSAI group; (D) Placebo plus NSAI group. OS, overall survival state; PFS, progression-free survival state; PD, progression disease state.

Supplementary Figure S6. Probability sensitivity analysis scatter plot

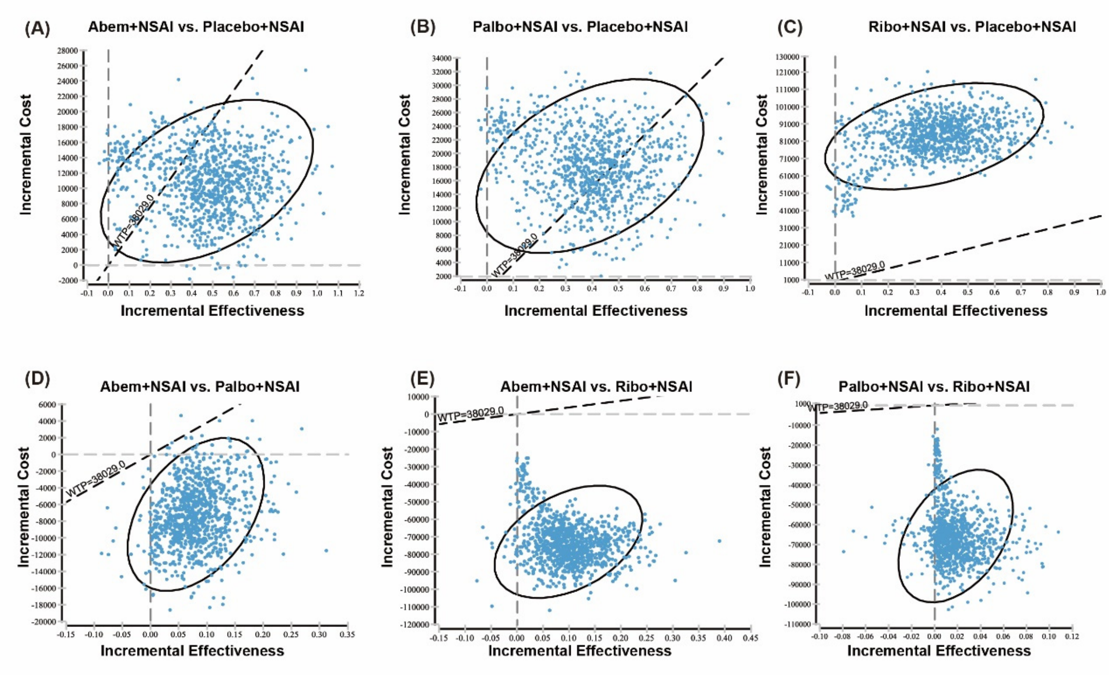

Each point in the diagram represents a simulation result of 10,000 Monte Carlo simulation. Ellipse represent the 95% CI and dotted line represent WTP ($38 029/QALY). Points to the right of the dotted line are considered cost-effective.

(A) Abem+ NSAI vs. Placebo+ NSAI;

(B) Palbo+ NSAI vs. Placebo+ NSAI;

(C) Ribo+ NSAI vs. Placebo +NSAI;

(D) Abem+ NSAI vs. Palbo +NSAI;

(E) Abem+ NSAI vs. Ribo+ NSAI;

(F) Palbo+ NSAI vs. Ribo+ NSAI.

WTP, willingness-to-pay; Palbo, palbociclib, Ribo, ribociclib, ABE, Abem, LET, letrozole, NSAI, letrozole/anastrozole.
